# Autophagy mediates ER stress and inflammation in *Helicobacter pylori*-related gastric cancer

**DOI:** 10.1080/19490976.2021.2015238

**Published:** 2021-12-29

**Authors:** M.C. Mommersteeg, I. Simovic, B. Yu, S.A.V. van Nieuwenburg, I, M.J. Bruno, M. Doukas, E.J. Kuipers, M.C.W. Spaander, M.P. Peppelenbosch, N. Castaño-Rodríguez, G.M. Fuhler

**Affiliations:** a Department of Gastroenterology and Hepatology, Erasmus University Medical Center, Rotterdam, The Netherlands; b School of Biotechnology and Biomolecular Sciences, Unsw, Sydney, Australia; c Department of Pathology, Erasmus University Medical Center, Rotterdam, Netherlands

**Keywords:** Intestinal metaplasia, atrophic gastritis, gastric cancer, *Helicobacter pylori*, autophagy, ER stress, inflammation, ATG16L1

## Abstract

Autophagy is a cellular degradation mechanism, which is triggered by the bacterium *Helicobacter pylori*. A single nucleotide polymorphism (SNP) in the autophagy gene *ATG16L1* (rs2241880, G-allele) has been shown to dysregulate autophagy and increase intestinal endoplasmic reticulum (ER) stress. Here, we investigate the role of this SNP in *H.* *pylori*-mediated gastric carcinogenesis and its molecular pathways. *ATG16L1* rs2241880 was genotyped in subjects from different ethnic cohorts (Dutch and Australian) presenting with gastric (pre)malignant lesions of various severity. Expression of GRP78 (a marker for ER stress) was assessed in gastric tissues. The effect of *ATG16L1* rs2241880 on *H.* *pylori*-mediated ER stress and pro-inflammatory cytokine induction was investigated in organoids and CRISPR/Cas9 modified cell lines. Development of gastric cancer was associated with the *ATG16L1* rs2241880 G-allele. Intestinal metaplastic cells in gastric tissue of patients showed increased levels of ER-stress. *In vitro* models showed that *H.* *pylori* increases autophagy while reducing ER stress, which appeared partly mediated by the *ATG16L1* rs2241880 genotype. *H.* *pylori*-induced IL-8 production was increased while TNF-α production was decreased, in cells homozygous for the G-allele. The *ATG16L1* rs2241880 G-allele is associated with progression of gastric premalignant lesions and cancer. Modulation of *H.* *pylori*-induced ER stress pathways and pro-inflammatory mediators by *ATG16L1* rs2441880 may underlie this increased risk.

## Introduction

*Helicobacter pylori* is recognized as a class 1 carcinogen associated with gastric cancer.^1^ This pathogen contributes to the large majority of intestinal-type gastric adenocarcinomas (GC), which affects over 1 million patients each year.^2^ While the association between *H. pylori* infection and GC risk is irrefutable, the exact causative mechanism remains unclear. It is generally accepted that *H. pylori*-induced inflammatory processes resulting in morphological alterations of the gastric mucosa are a contributing factor, as GC is commonly preceded by premalignant lesions. The first lesion to be recognized is atrophic gastritis characterized by the disappearance of parietal cells and gland tissue. This can be followed by development of intestinal metaplasia (IM), defined by colonic features such as goblet cells and expression of intestinal markers.^3,4^ Finally, progression into malignant lesions such as dysplasia and GC can occur.

Despite the causative relationship between *H. pylori* virulence factors and gastric carcinogenesis, not everyone with *H. pylori* or gastric premalignant lesions will eventually develop GC. Therefore, interest in host factors influencing the gastric environment and gastric carcinogenesis has taken flight.^5^ One of these host factors potentially associated with development of GC is a single nucleotide polymorphism (SNP) in the gene Autophagy Related 16 Like 1 (*ATG16L1*).

*ATG16L1* rs2241880 denotes a threonine to alanine change at position 300 (in which the A-allele corresponds to threonine or wild type and the G-allele corresponds to alanine or mutant), which causes a defect in autophagy,^6,7^ a cellular process in which un- or misfolded proteins from the endoplasmic reticulum (ER) are recycled. Importantly, autophagy can also be directed against intracellular pathogens and thus serves as an innate immune defense mechanism. Consequently, bacterial clearance is affected by *ATG16L1* rs2241880^7^. However, the role of autophagy in *H. pylori* infection appears to be complex as *H. pylori* may induce autophagy to promote its replication.^8–10^ Another key role for autophagy in the response to infection is the modulation of immune cell effectors, cytokine production and the control of inflammation.^11^ However, the exact ramifications of the autophagic abnormalities evoked by *ATG16L1* rs2241880 on the innate immune response to *H. pylori* infection are unknown. This is further muddled by current conflicting findings from studies investigating the role of *ATG16L1* rs2241880 and the risk of GC development in European and East Asian populations.^12,13^

Autophagy itself can be either anti- or pro-carcinogenic depending on disease stage and metabolic requirements.^14^ Many tumor types show enhanced protein synthesis, which can cause ER stress by overloading of the ER with un-foldable proteins. Compensatory upregulation of the autophagic process to reduce ER stress is thus often observed in tumors.^15,16^ However, when autophagy fails to be properly activated, as is the case for *ATG16L1* rs2241880, ER stress may be enhanced. Alternative ER stress alleviation comes from the unfolded protein response (UPR), aimed at temporarily blocking protein synthesis,^17^ and ER-stress markers and the UPR are indeed known to be induced in GC.^18^ The most important ER-stress biomarker, the unfolded protein chaperone GRP78, was associated with poor patient survival in a GC cohort comprising 328 patients.^19^

Autophagy, *ATG16L1* rs2241880, inflammation and ER stress have all been previously linked to *H. pylori* infection and GC, however, the interplay between these factors in gastric carcinogenesis is unclear. In this study, we aimed to investigate these processes in both gastric premalignant lesions and GC, in diverse ethnic groups.

## Results

### *ATG16L1* rs2241880 G allele is associated with gastric cancer in an Australian population and this risk increases synergistically with *Helicobacter pylori* infection

To substantiate the role of *ATG16L1* rs2241880 in the GC cascade, this polymorphism was first investigated in Australian Caucasians presenting with GC (n = 117) compared to healthy controls (n = 232). The minor allele within this cohort is the G allele, therefore, analyses were conducted with the A allele as reference. Both G allele (OR: 1.42, 95% CI: 1.04–1.95) and mutant homozygosity (GG) (OR: 1.96, 95% CI: 1.03–3.69) were found to be risk factors for GC development in this population (Tables 1 and 2).

**Table 1.** Association between *ATG16L1* rs2241880 alleles and risk of gastric precancerous lesions and gastric cancer in two human populations

Population	Disease	Mutant Allele	Allele frequencies				Allele analysis		
			Cases		Controls				
			A	G	A	G	OR	95% CI	*P*
Dutch	IM	A	234	206	4337	5197	**1.36**	**1.12–1.65**	**0.0017**
Australian	GC	G	102	132	243	221	**1.42**	**1.04–1.95**	**0.0305**

**Table 2.** Association between *ATG16L1* rs2241880 genotypes and risk of gastric precancerous lesions and gastric cancer in two human populations

Population	Disease	Mutant Genotype	Genotype frequencies						Genotype analysis											
			Cases			Controls			WT Homo vs Mut Homo			WT Homo vs Hetero			Dominant model			Recessive model		
			AA	AG	GG	AA	AG	GG	OR	95% CI	*P*	OR	95% CI	*P*	OR	95% CI	*P*	OR	95% CI	*P*
Dutch	IM	AA	56	122	42	975	2387	1405	**1.92**	**1.28–2.89**	**0.0017**	**1.71**	**1.20–2.47**	**0.0028**	**1.77**	**1.26–2.49**	**0.0008**	1.33	0.97–1.81	0.0743
Australian	GC	GG	24	54	39	65	113	54	**1.96**	**1.03–3.69**	**0.0427**	1.29	0.73–2.33	0.3959	1.51	0.90–2.54	0.1526	1.65	1.02–2.71	0.0543

Since infection with *H. pylori* is central to GC etiology, we further evaluated the existence of a potential synergistic relationship between the polymorphism and infection on the risk of GC. This analysis showed that individuals who carried the G allele and were infected with *H. pylori* were the most at risk of GC as compared to those harboring the AA genotype, showing a substantial OR of 3.25 (95% CI: 1.25–8.09) (Table 3).

**Table 3.** Joint effect analysis of *Helicobacter pylori* infection and *ATG16L1* rs2241880 polymorphism on gastric cancer risk in an Australian Caucasian population

*H. pylori* status	Genotype	OR	95% CI	*P*
(-)	AA	1		
(-)	G carrier	1.11	0.38–3.07	> 0.9999
(+)	AA	2.12	0.69–5.73	0.2058
**(+)**	**G carrier**	**3.25**	**1.25–8.09**	**0.0210**

### *ATG16L1* rs2241880 A allele is associated with mild gastric premalignant lesions in a Dutch population

We then investigated whether *ATG16L1* rs2241880 is associated with the GC precursor lesion IM in a Dutch IM cohort (n = 308) compared to the population-based cohort of the Rotterdam study I (n = 4542).^20^ The minor allele within this cohort is the A allele, therefore, analyses were conducted with the G allele as reference. A significant increased risk of developing premalignant lesions was found for individuals with an A allele (A allele – OR 1.36, 95% CI 1.12–1.65, AA genotype – OR 1.92, 95% CI 1.28–2.89, AG genotype – OR 1.71, 95% CI 1.20–2.47, dominant model – OR 1.77, 95% CI 1.26–2.49) (Tables 1 and 2). Because it is the *ATG16L1* rs2241880 G allele that has been previously associated with GC,^12^ as also evidenced in our current Australian Caucasian cohort, we further subdivided patients into those whose gastric premalignant lesions were more severe (defined as an OLGIM stage III or IV) and those with mild premalignant lesions (OLGIM I or II). Interestingly, within this population we found that the A allele is only associated with an increased risk of mild premalignant lesions (A allele – OR 1.49, 95% CI 1.15–1.92, AA genotype – OR 2.48, 95% CI 1.38–4.52 and AG genotype – OR 2.32, 95% CI 1.38–3.84) as no significance was reached in either allele or genotype analyses of severe premalignant lesions (Tables 4 and 5).

**Table 4.** Association between *ATG16L1* rs2241880 alleles and the risk of mild or severe intestinal metaplasia (OLGIM) in a Dutch population

Disease subgroup	Mutant Allele	Allele frequencies				Allele analysis		
		Cases		Controls				
		G	A	G	A	OR	95% CI	*P*
Mild IM (OLGIM I/II)	A	107	133	5197	4337	**1.49**	**1.15–1.92**	**0.0025**
Severe IM (OLGIM III/IV)	A	99	101	5197	4337	1.22	0.93–1.61	0.1729

**Table 5.** Association between *ATG16L1* rs2241880 genotypes and the risk of mild or severe intestinal metaplasia (OLGIM) in a Dutch population

Disease	Genotype frequencies						Genotype Analysis											
	Cases			Controls			AA vs GG			AG vs GG			Dominant model			Recessive model		
	GG	AG	AA	GG	AG	AA	OR	95% CI	*P*	OR	95% CI	*P*	OR	95% CI	*P*	OR	95% CI	*P*
Mild IM (OLGIM I/II)	18	71	31	1405	2387	975	**2.48**	**1.38–4.52**	**0.002**	**2.32**	**1.38–3.84**	**0.0011**	**2.37**	**1.42–3.98**	**0.0003**	1.36	0.88–2.04	0.1693
Severe IM (OLGIM III/IV)	24	51	25	1405	2387	975	1.50	0.86–2.63	0.1867	1.25	0.76–2.04	0.3998	1.32	0.84–2.09	0.2676	1.30	0.81–2.02	0.2615

### Functional consequences of *ATG16L1* rs2241880 on gastric ER stress

**Figure 1.**
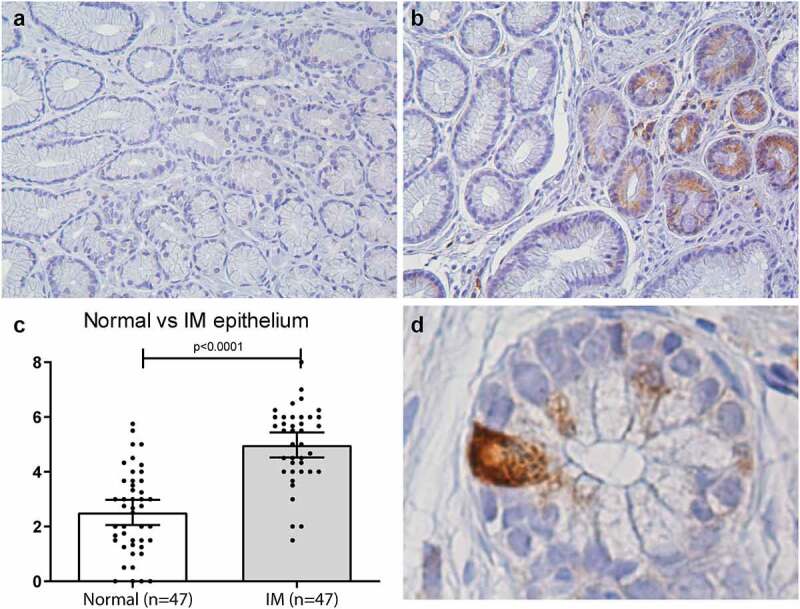
**ER stress (GRP78) is increased in gastric IM tissues (taken by forceps biopsy from the gastric antrum)**. Representative pictures of antral biopsies (n = 23) immunohistochemically stained for GRP78 (brown). (a) Normal epithelium of the gastric antrum, showing no IM. (b) Representation of a biopsy including mixed normal epithelium (left) and IM (right). (c) Quantitative analyses showing that IM tissues of the gastric antrum express more GRP78 than the normal epithelium as measured by Allred score, Statistical analysis was carried out using parametric t-test (two-tailed) with errors bars representing SEM (d) Magnification of individual crypt showing strong GRP78 staining located mainly in an intestinal Paneth cell, representing an IM patch within the biopsy. IM: intestinal metaplasia

**Figure 2.**
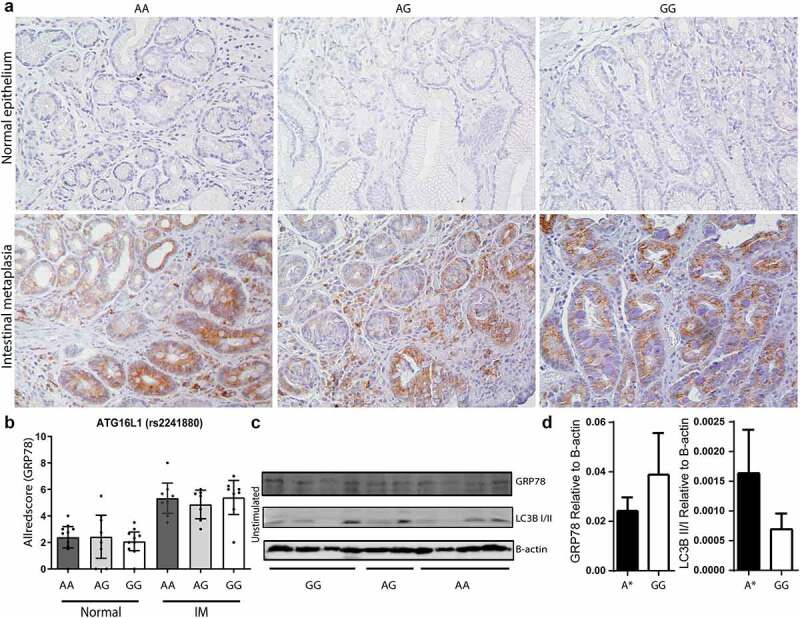
**ER stress (GRP78) in gastric IM epithelium is not influenced by *ATG16L1* rs2241880 status**. (a) Representative pictures of normal epithelium (upper panels) and intestinal metaplastic tissues (IM, lower panels), immunohistochemically stained for GRP78. Representative examples of *ATG16L1* rs2241880 AA, AG and GG carriers are shown for both normal and IM tissues. (b) Quantification of Allred scores separated according to *ATG16L1* rs2241880 genotype. Statistical analysis was carried out using parametric t-test (two-tailed) with errors bars representing SEM, (c) Western blot analysis of unstimulated (basal) GRP78 levels and LC3B expression in *H. pylori* stimulated organoids derived from intestinal metaplasia (IM) patients. (d) Quantification of Western blot analysis of GRP78 expression and LC3BII/I ratio normalized against the expression of β-actin in *H. pylori* stimulated organoids derived from IM patients. A* indicates AA and AG allele carriers. IM: intestinal metaplasia, AA; wild-type homozygote, AG; heterozygote, GG; mutant homozygote

Previous studies have shown that reduced autophagy in *ATG16L1* rs2241880 G-allele carrying cells may lead to increased levels of ER stress in intestinal epithelia.^21^ Therefore, we next investigated the functional consequence of this locus on GRP78 expression in antral biopsies of patients diagnosed with gastric premalignant lesions (from the Dutch cohort). Figure 1(a-b) shows that ER stress was indeed present in antral tissues of IM patients, and was mainly limited to the IM crypts (quantified in Figure 1(c)). Of note, the cell-type staining most positive for GRP78 are Paneth cells, which is in line with previous data showing that these cells are susceptible to ER stress (see example in Figure 1(d)).^7^ However, when patients were stratified according to their genotype, no association was observed between the *AGT16L1* rs2241880 status and GRP78 expression in the antral biopsies (Figure 2(a-b)). Furthermore, we saw no significant differences in constitutive GRP78 expression between organoids (derived from IM patients from the Dutch cohort) carrying different variants of this *ATG16L1* locus (Figure 2(c-d)). Thus, these data suggest that ER stress levels in gastric epithelium *per se* are not affected by *ATG16L1* rs2241880 status.

### *H. pylori* reduces ER stress levels in gastric epithelium, while enhancing autophagy

**Figure 3.**
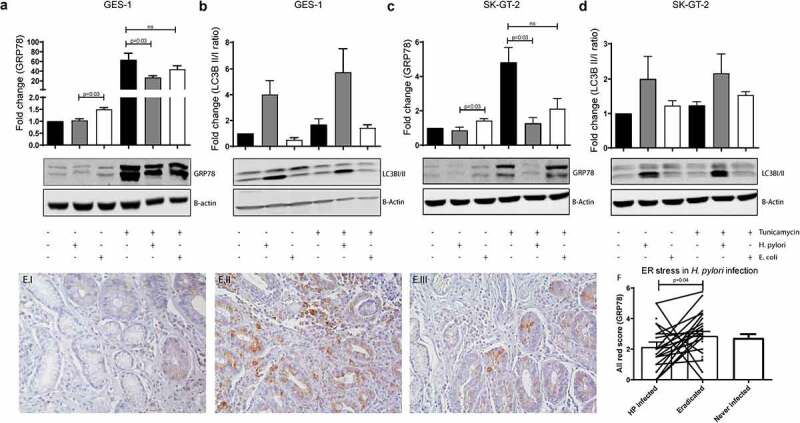
***Helicobacter pylori* reduces ER stress levels in IM tissues**. (a-d) GRP78 (a, c) and LC3II expression (b, d) determined by Western blot analysis in GES-1 normal gastric epithelial cells (a, b) and SK-GT-2 gastric cancer cell line (c, d). Representative example as well as quantification of 4 individual experiments is shown. GRP78 expression and LC3B II/I ratio relative to B-actin and normalized against unstimulated sample, are shown. Statistical analysis was carried out using Mann-Whitney test (two-tailed) with errors bars representing SEM (e) Representative images of GRP78 staining of antral biopsies from a patient with active infection with *H. pylori* **(E.I)**, the same patient 1 year after eradication of *H. pylori* (HP) (**E.II**), and an antral biopsy from a second patient who has not encountered *H. pylori* **(E.III)**. (f) Bar graph of quantified Allred scores of the average of biopsies stained for GRP78 during infection (n = 24), after eradication (n = 23) and in patients who have never been infected (n = 23). IM: intestinal metaplasia

As we observed enhanced ER stress levels in IM crypts, but no direct association with *ATG16L1* rs2241880 status in either IM biopsies or organoids, we wondered whether ER stress would be modulated by *H. pylori* infection. Therefore, we examined the effect of *H. pylori* on the inter-relationship between autophagy and ER stress in gastric cell lines. GES-1 and SK-GT-2 cell lines were stimulated with *H. pylori* in the absence or presence of tunicamycin, an antibiotic which induces accumulation of unfolded proteins.^22^ Stimulation with *H. pylori* for 16 hours did not affect GRP78 expression in GES-1 cells (Figure 3(a)) or SK-GT-2 cells (Figure 3(c)), however, in cells experiencing ER stress induced by tunicamycin, *H. pylori* but not *E. coli*, significantly reduced GRP78 levels. It is important to note that GRP78 levels were significantly increased in cells stimulated with *E. coli* when compared to cells stimulated with *H. pylori*, also suggesting that reduction of GRP78 is much less efficiently induced by other bacteria. We next investigated whether *H. pylori* also affects ER stress levels *in vivo*. To this end, we compared GRP78 levels in patients with an active infection at time of biopsy sampling (n = 23) and biopsies of these same individuals after eradication treatment. ER stress levels were indeed significantly lower at time of infection with *H. pylori* (Figure 3(e-f)). Upon eradiation of *H. pylori*, GRP78 increased to levels indistinguishable from patients who have never been infected with *H. pylori* (n = 24). These data suggest that GRP78 levels in IM crypts are negatively affected by the presence of *H. pylori*.

We hypothesized that *H. pylori* might induce autophagy and thereby resolve the ER stress induced by tunicamycin. Therefore, we investigated the activation of autophagy by determining the ratio of conjugated to unconjugated LC3B, an established autophagy marker. Interestingly *H. pylori*, but not *E. coli*, indeed appears to increase the LC3B II/I ratio in both unstimulated and tunicamycin-stimulated GES-1 (Figure 3(b)) and SK-GT-2 cells (Figure 3(d)). This effect appears to be specific to *H. pylori* as well as gastric epithelial cells, as no effect of *H. pylori* on ER stress was observed in either colon cancer or esophageal cell lines (**Supplementary Figure S1**).

### *H. pylori*-induced ER stress reduction may be correlated to *ATG16L1* rs2241880 status

**Figure 4.**
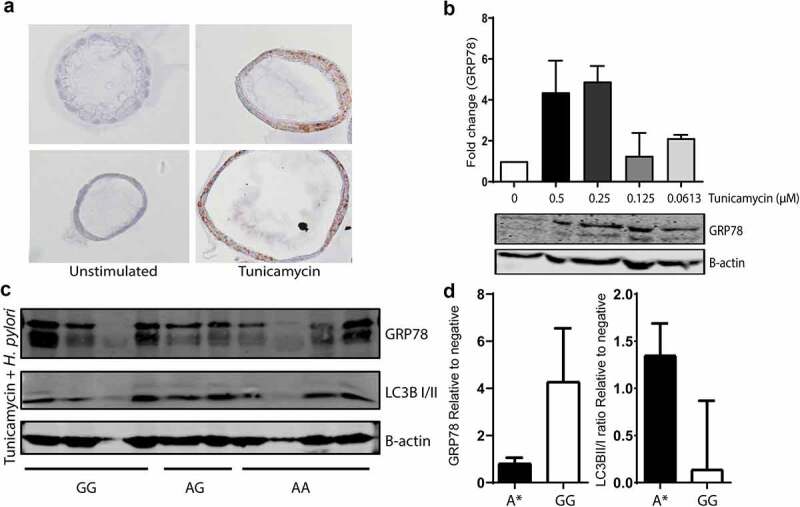
***Helicobacter pylori*-induced ER stress reduction may be correlated to *ATG16L1* rs2241880 status**. (a) Immunohistochemical staining for GRP78 on IM organoids from the same patient (AA) and same passage number, stimulated with tunicamycin for 16 hours. (b) Western blot analysis for GRP78 of IM organoid samples (n = 3, GT: AA) stimulated with increasing concentrations of tunicamycin for 16 hours. (c) Western blot analysis for GRP78 and LC3B of IM organoid samples from patients with differing *ATG16L1* rs2241880 genotypes. (d) Bar graph of quantified Western blot analysis for GRP78 and LC3B II/I ratio relative to β-actin and normalized against unstimulated samples. Statistical analysis was carried out using Mann-Whitney test (two-tailed) with errors bars representing SEM. A* denotes AA/AG genotypes. IM: intestinal metaplasia, AA; wild-type homozygote, AG; heterozygote, GG; mutant homozygote

To assess the effect of *ATG16L1* rs2241880 on *H. pylori*-reduced ER-stress, we studied gastric organoids individually derived from 10 IM patients with differing genotypes (4 GG, 3 AG, 4 AA). First, we verified that ER stress (basal levels of which are low in gastric organoids, see Figures 2(c-d) and 4a) could be induced by tunicamycin (Figure 4(a-b)). ER-stressed cells were subsequently treated with *H. pylori*. A non-significant increase in GRP78 expression (*p* = .16) was seen in organoids from G-allele homozygous individuals (Figure 4(c-d)). Conversely, a suggestive reduction of the LC3B II/I ratio in samples from patients with a GG genotype was seen (*p* = .062) (Figure 4(c-d)). These data demonstrate a trend toward reduced autophagy induction by *H. pylori* in individuals carrying the *ATG16L1* rs2241880 G-allele.

### *ATG16L1* rs2241880 affects IL-8 production in gastric epithelial cells, but not in immune cells

We next sought to investigate other molecular consequences of carrying *ATG16L1* rs224180. For this, we used the *ATG16L1* rs224180 AG genotype-carrying AGS cell line to generate both *ATG16L1* rs224180 AA and GG daughter cell lines by CRISPR/Cas9 genome editing. We first assessed the autophagic flux in these gastric epithelial cells using p62 turnover assays. As expected, starvation induced autophagy while treatment with chloroquine inhibited this pathway (**Supplementary Figure S2)**. Importantly, autophagy was impaired in GG-carrying cells when compared to AG- and AA-carrying cells, with increased accumulation of p62 with chloroquine and decreased degradation of p62 with starvation (**Supplementary Figure S2**), confirming our suggestive findings in gastric organoids generated from IM patients.

**Figure 5.**
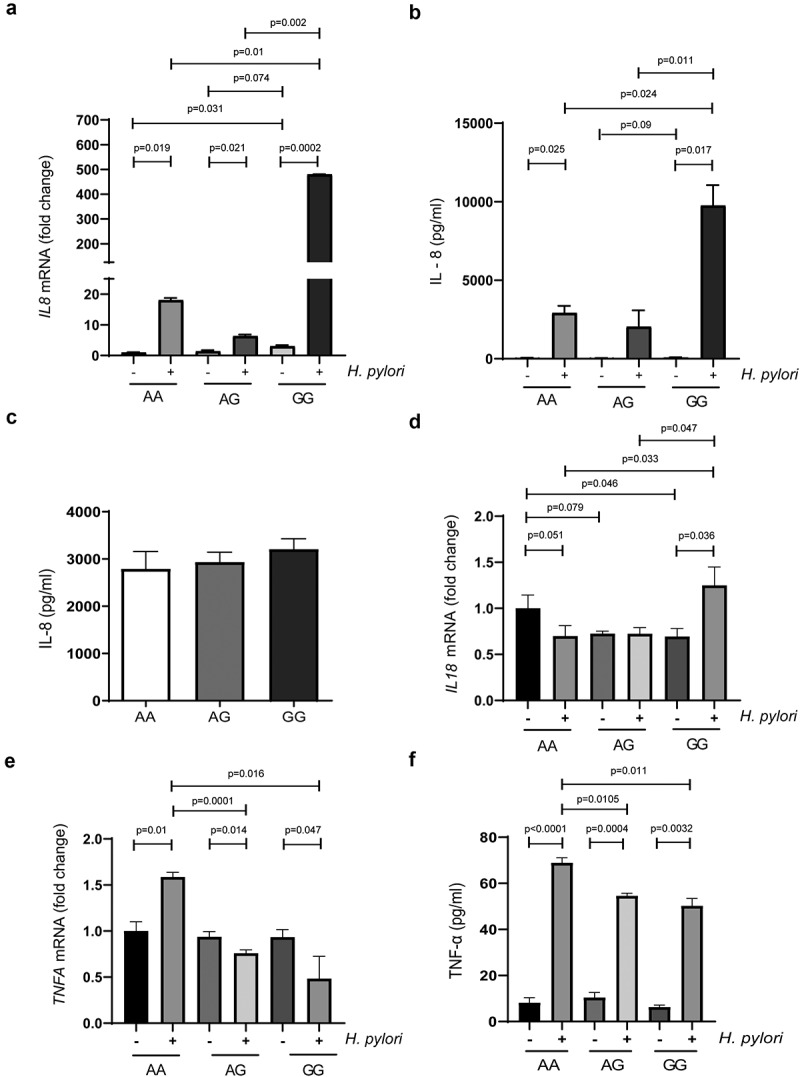
***ATG16L1* rs2241880 status does not affect IL-8 production in immune cells but modulates IL-8 and TNF-α production in AGS cells upon infection with *Helicobacter pylori***. (a) Expression of *IL8* mRNA in *H. pylori* strain GC026-challenged edited and non-edited AGS cells. (b) Concentration of IL-8 in *H. pylori* strain GC026-challenged edited and non-edited AGS cells. (c) Isolated PBMCs derived from healthy Dutch volunteers (AA n = 4; AG n = 6; GG n = 4) release substantial amounts of IL-8 after 2 hours of stimulation with *H. pylori*, but this is not affected by donor *ATG16L1* rs2241880 status. (d) Expression of *IL18* mRNA in *H. pylori* strain GC026-challenged edited and non-edited AGS cells. (e) Expression of *TNFA* mRNA in *H. pylori* strain GC026-challenged edited and non-edited AGS cells. (f) Concentration of TNF-α in *H. pylori* strain GC026-challenged edited and non-edited AGS cells. Experiments with the AGS cells were conducted in triplicates. Statistical analysis was carried out using parametric t-test (two-tailed) with errors bars representing SEM. AA; wild-type homozygote, AG; heterozygote, GG; mutant homozygote

IL-8 production contributes to gastric carcinogenesis through inflammation-mediated tissue destruction,^23^ therefore, we investigated the effect of the three *ATG16L1* rs224180 genotypes on IL-8 production in these cells. Across all three genotypes we detected a significant upregulation in the expression of *IL8* following infection with *H. pylori*, the most dramatic of which was observed in GG-carrying cells, which demonstrated a 480-fold change (*p* = .0002) (Figure 5(a)). The AG- and AA-carrying cells in comparison elicited a modest upregulation of 6- and 18-fold, respectively. Importantly, at basal levels (i.e. no exposure to *H. pylori*), expression of *IL8* mRNA was 3-fold higher in the GG-carrying cells compared to the AA-carrying cells (*p* = .0310). The same phenomenon was also suggested when comparing with AG-carrying cells (*p* = .074). The above findings were mostly corroborated at the protein level (Figure 5(b)) and support the notion that disruptive autophagy promotes an increased IL-8 response in epithelial cells upon infection.^24,25^

We then turned our attention to the effect of *ATG16L1* genotype status on *H. pylori*-induced IL-8 production by PBMCs from healthy individuals with differing genotypes (4 AA, 6 AG and 6 GG). While IL-8 was clearly induced upon *H. pylori* stimulation, no difference in IL-8 production was observed between different genotypes (Figure 5(c)). This is in line with data showing no differences in PBMC cytokine production in IBD patients with either the *ATG16L1* GG or AA phenotype,^26^ and suggests that immune cells are not affected by rs2241880 status upon *H. pylori* stimulation.

### *ATG16L1* rs2241880 modulates the production of other cytokines in gastric epithelial cells

To further explore the extent of the role of *ATG16L1* rs2241880 on the innate immune response to *H. pylori* infection in gastric epithelial cells, we investigated a profile of both pro- and anti-inflammatory cytokines including TNF-α, IL-1β, IL-10, IL-17A and IL-18.

Accumulating evidence suggests TNF-α is a key player in tumorigenesis,^27^ thus, we sought to evaluate its impact in *H. pylori-*driven gastric carcinogenesis and its effect under *ATG16L1* rs2441880. At the mRNA level, we observed that *H. pylori-*infected AA-carrying cells exhibited a significantly higher fold change in *TNFA* expression compared to infected G-allele carrying cells (Figure 5(e)). Interestingly, upon infection, the G allele-carrying cells experienced a significant downregulation in *TNFA* expression, with the greatest fold change decrease demonstrated in homozygotes (0.48, *p* = .047). We legitimize these findings at the protein level (Figure 5(f)), where infected AA-carrying cells demonstrate the highest release of TNF-α, while the infected AG- and GG- carrying cells show a significantly lower yield (both *p = *.011). These results are also in-line with previous studies which demonstrate that the GG genotype significantly impacts the production of TNF-α in PBMC exposed to various bacteria and *Candida.^28,^^29^*

The potent pro-inflammatory cytokine IL-18 is implicated in the pathogenesis of GC via its modulation of tumor cell survival and expression of metastasis-associated genes.^30^ Upon infection, we observed significant *IL18* upregulation in our GG-carrying cells (Figure 5(d), *p = *.036), which is also significantly higher when compared across the other genotypes. We attempted to validate these findings at the protein level, however, production of mature IL-18 in these AGS cells was extremely limited and statistical analysis determined no significant differences in expression of IL-18 across the different genotypes (**Supplementary Figure S5)**.

A disadvantage of our employment of a gastric epithelial cell line was the limited expression of some of our immune targets. This was the case for IL-1β, IL-10 and IL-17A, which revealed non-amplifiable mRNA levels in AGS cells, as well as undetectable supernatant protein levels using a multiplex ELISA (not shown).

## Discussion

Here, we investigated the role of the autophagy gene *ATG16L1* in the progression of *H. pylori*-mediated gastric carcinogenesis. In carcinogenesis, autophagy may be exploited as a protective mechanism against apoptosis-inducing drugs or be downregulated in an effort to dysregulate cellular replication.^31,32^ In general, it is suggested that defective autophagy promotes tumorigenesis,^33^ with mutations in several autophagy-related genes occurring in GC.^34,35^ In addition to somatic mutations, germline genetic variants affecting the autophagy pathway may also contribute to gastric carcinogenesis. SNPs in genes regulating the autophagy lysosome pathway were significantly associated with GC in GWAS analyses.^36^ While genome-wide significance for any individual gene was not reached, the G-allele of *ATG16L1* rs2241880, which renders autophagy less efficient, was associated with an increased risk for GC and *H. pylori* infection in a Chinese population.^8,13^ These findings are also consistent with a study demonstrating a positive correlation between mutant homozygosity (GG) and susceptibility to *H. pylori* infection in 162 Scottish and 483 German subjects.^8^ However, others were unable to find associations with *ATG16L1* rs2241880,^37,38^ indicating that the exact role of host factors in *H. pylori* infection and subsequent gastric carcinogenesis remains elusive. In the current study, the G allele was associated with risk of GC within Australian Caucasians, and this risk was further exacerbated in *H. pylori*-infected individuals. GC is preceded by several premalignant lesions, which themselves are positively associated with *H. pylori* infection. Thus, we expected *ATG16L1* rs2241880 to also be associated with these lesions. Interestingly, data from the Dutch population suggest that the G allele is not associated with development of early premalignant lesions. Overall, these results suggest that mild (OLGIM I–II) and severe (i.e. OLGIM stage III–IV, dysplasia and GC) disease stages are not similarly affected by *ATG16L1* rs2241880. However, an important variable that was not addressed here was the presence and diversity of the infecting *H. pylori* strains. Virulence of the infecting *H. pylori* strains can influence disease outcome and severity, and within Scottish and German populations, *ATG16L1* rs2241880 homozygosity conferred more susceptibility to *vacA s1m1* strains compared to the *s1m2* strains.^8^

The *ATG16L1* rs2241880 G-allele is thought to impair autophagy, which in itself is required for reducing cellular ER-stress.^39–41^ As such, *ATG16L1* rs2241880 is associated with enhanced accumulation of unfolded proteins and ER stress markers in gastrointestinal disease. For example, in Crohn’s disease, where *ATG16L1* rs2241880 represents a widely established genetic risk factor,^7,42^ the G-allele is associated with increased small intestinal ER-stress and GRP78 expression, most notably in intestinal Paneth cells.^7^ Interestingly, Paneth cells also appear during the development of complete intestinal metaplasia, and we found that ER-stress accumulation was most noticeable in these Paneth cells within IM tissues, although this was not associated with *ATG16L1* rs2241880 status *per se*. We show that *H. pylori* reduces ER stress levels *in vitro* and *ex vivo*, possibly by induction of the autophagy pathway, and that this may be modulated by *ATG16L1* rs2241880. This is in line with reports demonstrating that *H. pylori* activates and uses the autophagy pathway for its replication.^43,44^ However, this does raise the question as to why ER stress levels in general are upregulated in IM regions of the antrum. Even though IM may be caused by *H. pylori*-induced inflammation, the bacteria are unable to survive in the metaplastic crypts.^45^ Thus, ER stress induction in IM *per se* may be *H. pylori-*independent, which may partly explain why constitutive GRP78 levels in IM biopsies were *ATG16L1* rs224180-independent. While metaplasia is not considered malignant, the mutational burden is still increased when compared to the normal gastric epithelium,^46^ which in turn can increase ER stress by overloading the folding capacity of the protein synthesis apparatus, in particular in cell types with high-protein production such as secretory granule-rich Paneth cells.^47^

We next sought to elucidate the molecular interactions between *ATG16L1* rs2241880 and *H. pylori*-associated gastric carcinogenesis. Matching SNPs to their relevant cell type is imperative in order to understand their functional consequence.^48^ Previous studies on *ATG16L1* silencing have demonstrated that the subsequent disruptive autophagy is associated with an elevated production of the important pro-inflammatory cytokine IL-8 in epithelial cells exposed to *Shigella* and *Haemophilus parasuis* SH0165.^24,25^ We demonstrated that while *H. pylori* induced IL-8 production in PBMCs, this was not affected by *ATG16L1* rs2241880 status. In contrast, IL-8 production was significantly enhanced in GC cells harboring the GG-genotype. These data suggest that rs2241880 has a predominant effect on the epithelial compartment, although we cannot exclude the possibility that the diversity in other immune-modulatory SNPs present in PBMCs from different donors, but not in gene-edited AGS cells, masked the effect of *ATG16L1* rs2241880 on IL-8 production in these immune cells. Additionally, the effect may be cytokine-specific, as *ATG16L1* rs2241880 affects *C. albicans*-stimulated TNFα, but not IL-8, IL1β, IL-6 or INF-γ production in PBMCs.^28^ Nevertheless, an enhanced production of IL-8 by epithelial cells either constitutively or in response to *H. pylori* could contribute to an enhanced risk of carcinogenesis in GG-allele carriers, as IL-8 has been demonstrated to be an important factor in the sequence leading up to GC.^23,49–51^ It is of interest to note that IL-8-recruited granulocytes appear to play a role in IM and GC development, and that this may be independent of *H. pylori* infection.^52^ This is in line with our results showing that the absence of infection still elicits a phenotypic difference in IL-8 production between the three *ATG16L1* genotypes in AGS cells, which might independently contribute to carcinogenesis.

The role of the pro-inflammatory cytokine TNF-α in cancer is somewhat controversial with purported both anti- and pro-tumorigenic properties.^53^ Our data indicates that the *ATG16L1* rs2241880 G allele reduces TNF-α levels in gastric epithelial cells when infected with *H. pylori*, while the A allele reports an increase. TNF-α production was also found to be decreased in PBMCs from patients harboring the GG genotype when stimulated with EIEC, *Listeria monocytogenes*, and *Salmonella typhimurium*, but not to other bacteria such as *Staphylococcus aureus* and *Mycobacterium avium paratuberculosis,^29^* suggesting that not only does *ATG16L1* rs2241880 modulate TNF-α but it functions in a pathogen-specific manner. Our data echoes previous results which highlighted that inhibition of autophagy in PBMC by 3 MA, an effect which may be mimicked by *ATG16L1* rs2214880, also lead to a decrease in TNF-α production upon stimulation with LPS.^54^ Interactions between TNF-α and autophagy are dynamic and two-way with both carrying the ability to regulate the other.^55^ Further studies should aim to elucidate the biological mechanisms underpinning the *ATG16L1* rs2241880 impact on TNF-α production.

*IL18* is constitutively expressed in epithelial cells and is synthesized as a precursor protein where the caspase-1 adaptor ASC (apoptosis-associated speck-like protein containing a caspase activation and recruitment domain) is required for maturation and cleavage of IL-18. While AGS cells can produce the precursor form of IL-18,^56^ they have been found to lack ASC and thus, are unable to produce mature IL-18.^57^ Our findings of limited IL-18 production in our AGS cells are, therefore, in concordance with the literature. We do show, however, that cells harboring the GG genotype have significantly increased levels of *IL18* mRNA, the effects of which in other cell types may confer a tumorigenic advantage for GC metastasis and evasion of immunosurveillance in the host.^58^

**Figure 6.**
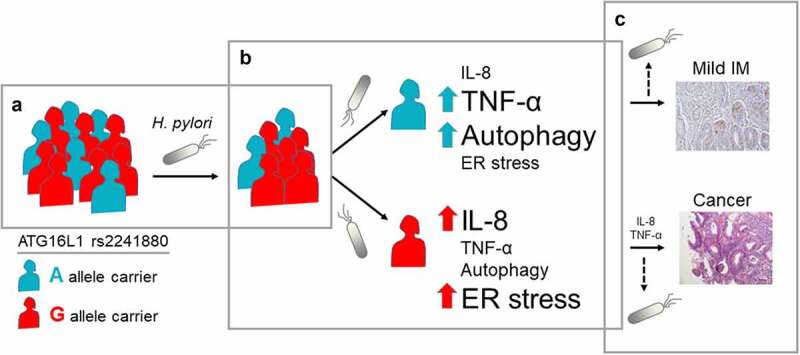
**Graphical representation of *ATG16L1* rs2241880 effect on gastric carcinogenesis**. (a) According to Correa’s cascade, gastric carcinogenesis starts by infection with *H. pylori*. While within the general population carriers of the *ATG16L1* rs2241880 G-allele are believed to be more prone to *H. pylori* infection, A-allele carriers are also infected. (b) In these A-allele carriers, *H. pylori*-evokes increased autophagy and TNF-α production, and less IL-8 production and endoplasmic reticulum (ER) stress, as compared to G-allele carriers, progressing only to mild intestinal metaplasia (IM). Enhanced IL-8 production and ER stress contribute to enhanced severity of gastric lesions in G-allele carriers. (c) While *H. pylori* is a leading cause in the carcinogenic process, this bacterium is generally not seen in established IM and gastric cancer. Thus, progression of IM to cancer may not rely solely on *H. pylori*-stimulated effects at these stages of the Correa sequence. As IM and cancer in themselves are prone to development of ER stress due to deregulated cellular processes, increased ER stress may no longer be dependent on *ATG16L1* rs2241880. However, *H. pylori-*independent epithelial IL-8 production is enhanced in *ATG16L1* rs2241880 G-allele carriers, which may further drive the carcinogenic process. AA; wild-type homozygote, AG; heterozygote, GG; mutant homozygote

From these data, a model appears (Figure 6) in which a reduced autophagic capacity conveyed by the *ATG16L1* rs2241880 GG-genotype contributes to gastric carcinogenesis through *H. pylori*-induced enhanced production of IL-8, decreased TNF-α levels, and enhanced ER-stress levels. In the absence of *H. pylori*, the carcinogenic process can be further driven in G-allele carriers by enhanced constitutive IL-8 levels. Overall, this work provides mechanistic insight into the role of genetic variants affecting autophagy in *H. pylori*-driven gastric carcinogenesis.

## Methods

### Population studies

#### Patient selection

Dutch patients presenting with gastric premalignant lesions were included from the ongoing prospective progression or regression of gastric premalignant lesions of the stomach (Proregal) cohort study^59,60^ (**Supplementary Table S1**). In total 308 patients were included for genetic analysis. For immunohistochemistry experiments, 47 patients were selected for whom a certified histological diagnosis of *H. pylori* was available or who were negative for *H. pylori* based on histology, serology and/or breath test. The study protocol was approved by the Institutional Review Board (MEC-2009-090), all patients signed an informed consent.

The Rotterdam Study is a population-based cohort of healthy individuals residing Rotterdam, details have been described previously.^20^ The cohort is representative of the Dutch population in a large city, comprising almost 6,500 healthy individuals aged between 45 and 75 years, with GWAS data available for n = 4542.

The Australian Caucasian study sample consisted of 117 non-cardia GC (NCGC) cases and 232 age-, gender- and ethnically-matched healthy controls (**Supplementary Table S1**). All subjects were of European ancestry (mainly from the United Kingdom, Greece, and Italy), aged between 40 and 70 years old, and resided in metropolitan Melbourne, Australia. Peripheral whole blood was collected between 1990 and 1994 as part of the ongoing Melbourne Collaborative Cohort Study (MCCS), where ethics approval was obtained by the Cancer Council Victoria (HREC 9500 and IEC 9001).

#### Human genomic DNA isolation and genotyping

For the Dutch IM population, DNA was isolated from whole blood using the Kleargene XL blood DNA extraction kit (LGC limited, Teddington, UK) and *ATG16L1* rs2241880 was determined using Polymerase Chain Reaction-Restriction Fragment Length Polymorphism (PCR-RFLP) (see supplementary materials for details and **Supplementary Figure S3** for examples). For the Australian population, genomic DNA was extracted from whole peripheral blood samples using the QIAamp Blood Mini Kit (Qiagen, Melbourne, Australia) and genotyped using matrix-assisted laser-desorption ionization time-of-flight (MALDI-TOF) mass spectrometry (MS) and the Agena Bioscience MassARRAY iPLEX assay (San Diego, USA) (see supplementary materials for details).

#### Statistical analysis

Deviation from Hardy-Weinberg equilibrium (HWE) for each population was tested using the chi-square (X^2^) goodness of fit test. Analyses were carried out using the Fisher’s exact probability test (two-tailed *P*-values), and the statistical package GraphPad Prism version 8.0.2 (GraphPad Software Inc., San Diego, USA). A *P-*value less than 0.05 was considered statistically significant while values less than 0.1 indicated trend.

### *In-vitro* studies

#### Cell lines

The GC epithelial cell lines SK-GT-2 and AGS, and immortalized gastric epithelial cell line GES-1 were cultured as described in supplementary material.

#### CRISPR/Cas9 Genome Editing

CRISPR/Cas9 genome editing technology was implemented to produce *ATG16L1* rs2241880 knock-in AGS cell lines representing all three genotypes of the polymorphism (AA, AG, GG). For details, see Supplementary Material. Genome editing validation was performed using qPCR as well as Sanger sequencing and fragment analysis.

#### Organoids

Stomach organoids from antrum and corpus were cultured as previously published,^61^ see Supplementary Material and **Supplementary Figure S4**. For stimulation experiments, organoids were first mechanically disrupted after which the cell suspension was equally divided and stimulated with *H. pylori*. All experiments using organoids were conducted from passages 3–6.

#### Immunohistochemistry

Immunohistochemistry was performed as described previously^7^ and positivity for GRP78 was quantified using the Allredscore.^62^ See supplement for details.

#### *Helicobacter pylori* culture and infection assays

*H. pylori* strain 43504 (*cagA^+^, vacA^+S1M1^*) was acquired from ATCC (Wesel, Germany). The *H. pylori* strain GC026 (*cag*A*^+^. cag*E^+^, *cag*L*^+^, cag*T^+^, *vacA^+^* ^s1m1^, *bab*A^+^, *oip*A^+^, *dupA*^+^, and *sabA*^+^) was previously isolated from a Malaysian GC patient.^63^ As control, *E. coli* (strain DH5α) was used. Methods for infection assays are detailed in supplementary material.

#### RT-PCR analysis

Quantitative gene expression analysis (qRT-PCR) was conducted for the target genes *TNFA, IL8, IL18, IL1B, IL10*, and *IL17A*, normalized to the house-keeping genes glyceraldehyde 3-phosphate dehydrogenase *(GAPDH)* and ribosomal protein lateral stalk subunit P0 (*RPLP0)*. All primers were supplied by Sigma-Aldrich (Sydney, Australia) (primer sequences and PCR protocols can be found in the **Supplementary Table S3** and **Table S4**). qRT-PCRs were performed independently for each gene using the SensiMix SYBR HI-ROX Kit (Bioline, Sydney, Australia) and the real-time PCR cycler Rotor Gene 6000 (Corbett Life Sciences, Sydney, Australia). The ^ΔΔ^CT method was implemented to quantify the relative gene expression of each cytokine in each subgroup.

#### Turnover assays and Western blot analysis

For turnover assays, *ATG16L1* rs2241880 knock-in AGS cell lines were subjected to serum starvation (Earle’s Balanced Salt Solution (Sigma) supplemented with sodium bicarbonate 2.2 g/L) or chloroquine (40uM) (Sigma) treatment, for 6 hours.

Western blot was performed as described previously.^64^ After incubation with the primary antibodies (GRP78 #3177S, LC3B-I/II #2775S, p62 #5114S; Cell Signaling Technology), membranes were incubated with IRDye antibodies (LI-COR Biosciences, Lincoln, NE, USA) or Goat Anti-Rabbit IgG (H + L)-HRP Conjugate (BioRad). Detection was performed using the Odyssey reader or the ImageQuant LAS 500 (GE Life Sciences, Uppsala, Sweden). See supplement for details.

#### Enzyme linked Immunosorbent Assay (ELISA)

Supernatant was collected from cell cultures, aliquoted and stored at −20°C until analysis. IL-8 was assessed either by singleplex ELISA (ready-set-go ELISA from eBioscience) or by a multiplex ELISA alongside targets IL-1β, IL-17A and IL-10 (Essential 4-Plex Human ProcartaPlex Panel 2 from ThermoFisher, Sydney, Australia). TNF-α and IL-18 were assessed using singleplex ELISAs (Abcam, Melbourne, Australia). All ELISAs were performed according to the manufacturer’s protocol.

Optical density (OD) measurement of plates was performed with either an infinite 200 pro ELISA reader (TECAN, Mannedorf, Switzerland) or the Dynamica HALO MPR-96 visible microplate reader (Dynamica, Gisbourne, VIC, Australia).

#### Statistical analysis

The data is represented by means ± SEM unless otherwise mentioned. Statistical analysis was performed using the student’s t-test, Mann Whitney U test or Fishers exact test (paired or unpaired, 95% confidence intervals, two tailed) using GraphPad (versions 5.0 or 8.0, GraphPad Inc, San Diego, CA, USA) and SPSS (Version 25, IBM, Armonk, United States).

## Supplementary Material

Supplemental Material

## Data Availability

Data supporting the findings of this study are available within the article and its supplementary materials.

## Author list

1. Michiel Christoffer Mommersteeg

Highest Academic Degrees: MD

Professional Titles: PhD candidate

Affiliations: Erasmus University Medical Center

Location of Affiliations: Doctor Molewaterplein 40, 3015 GD Rotterdam

Contributions: study concept and design, acquisition of data, analysis and interpretation of data, drafting manuscript, statistical analysis

1. Isidora Simovic

Highest Academic Degrees: B.Adv.Sc (Hons 1)

Professional Titles: PhD candidate

Affiliations: UNSW Sydney

Location of Affiliations: School of Biotechnology and Biomolecular Sciences, UNSW Sydney, Nsw 2052, Australia

Contributions: study concept and design, acquisition of data, analysis and interpretation of data, drafting manuscript, statistical analysis.

1. B. Yu

Highest Academic Degrees: MSc.

Professional Titles: PhD candidate

Affiliations: Erasmus University Medical Center

Location of Affiliations: Doctor Molewaterplein 40, 3015 GD Rotterdam

Contributions: Acquisition of data, administrative, technical, or material support

1. S.A.V. van Nieuwenburg

Highest Academic Degrees: MD

Professional Titles: PhD candidate

Affiliations: Erasmus University Medical Center

Location of Affiliations: Doctor Molewaterplein 40, 3015 GD Rotterdam

Contributions: Acquisition of data

1. M.J. Bruno

Highest Academic Degrees: PhD

Professional Titles: Professor

Affiliations: Erasmus University Medical Center

Location of Affiliations: Doctor Molewaterplein 40, 3015 GD Rotterdam

Contributions: Critical revision of the manuscript for important intellectual content, analysis and interpretation of data

1. M. Doukas

Highest Academic Degrees: PhD

Professional Titles: Pathologist

Affiliations: Erasmus University Medical Center

Location of Affiliations: Doctor Molewaterplein 40, 3015 GD Rotterdam

Contributions: Critical revision of the manuscript for important intellectual content, analysis and interpretation of data

1. E.J. Kuipers

Highest Academic Degrees: PhD

Professional Titles: Professor

Affiliations: Erasmus University Medical Center

Location of Affiliations: Doctor Molewaterplein 40, 3015 GD Rotterdam

Contributions: Critical revision of the manuscript for important intellectual content, analysis and interpretation of data

1. M.C.W. Spaander

Highest Academic Degrees: PhD

Professional Titles: Professor

Affiliations: Erasmus University Medical Center

Location of Affiliations: Doctor Molewaterplein 40, 3015 GD Rotterdam

Contributions: Critical revision of the manuscript for important intellectual content, acquisition of the data, analysis and interpretation of data, obtained funding

1. M.P. Peppelenbosch

Highest Academic Degrees: PhD

Professional Titles: Professor

Affiliations: Erasmus University Medical Center

Location of Affiliations: Doctor Molewaterplein 40, 3015 GD Rotterdam

Contributions: Study concept and design, critical revision of the manuscript for important intellectual content, analysis and interpretation of data, obtained funding

1. **Natalia Castaño-Rodríguez**

Highest Academic Degrees: PhD

Professional Titles: Cancer Institute NSW Research Fellow and UNSW Scientia Senior Lecturer

Affiliations: UNSW Sydney

Location of Affiliations: School of Biotechnology and Biomolecular Sciences, UNSW Sydney, NSW 2052, Australia

Contributions: Study concept and design, analysis and interpretation of data, drafting of the manuscript, critical revision of the manuscript for important intellectual content, obtained funding, study supervision

1. **Gwenny M. Fuhler**

Highest Academic Degrees: PhD

Professional Titles: Assistant- Professor

Affiliations: Erasmus University Medical Center

Location of Affiliations: Doctor Molewaterplein 40, 3015 GD Rotterdam

Contributions: Study concept and design, drafting of the manuscript, critical revision of the manuscript for important intellectual content, analysis and interpretation of data, obtained funding, study supervision

## References

[1] Schistosomes, liver flukes and Helicobacter pylori. IARC Working Group on the Evaluation of Carcinogenic Risks to Humans. Lyon. 7-14 June 1994 *IARC Monogr Eval Carcinog Risks Hum* 1994; 61 1–18. PMC7681621 7715068

[2] Bray F, Ferlay J, Soerjomataram I, Siegel RL, Torre LA, Jemal A. Global cancer statistics 2018: GLOBOCAN estimates of incidence and mortality worldwide for 36 cancers in 185 countries. CA Cancer J Clin. 2018;68(6):394–424. doi:10.3322/caac.21492. 30207593

[3] Goldenring JR, Nam KT. Oxyntic atrophy, metaplasia, and gastric cancer. Prog Mol Biol Transl Sci. 2010;96:117–131. 21075342 10.1016/B978-0-12-381280-3.00005-1 PMC4502917

[4] Park YH, Kim N. Review of atrophic gastritis and intestinal metaplasia as a premalignant lesion of gastric cancer. J Cancer Prev. 2015;20(1):25–40. doi:10.15430/JCP.2015.20.1.25. 25853101 PMC4384712

[5] Mommersteeg MC, Yu J, Peppelenbosch MP, Fuhler GM. Genetic host factors in Helicobacter pylori-induced carcinogenesis: emerging new paradigms. Biochim Biophys Acta Rev Cancer. 2018;1869(1):42–52. doi:10.1016/j.bbcan.2017.11.003. 29154808

[6] Cadwell K, Liu JY, Brown SL, Miyoshi H, Loh J, Lennerz JK, Kishi C, Kc W, Carrero JA, Hunt S, et al. A key role for autophagy and the autophagy gene Atg16l1 in mouse and human intestinal Paneth cells. Nature. 2008;456(7219):259–263. doi:10.1038/nature07416. 18849966 PMC2695978

[7] Deuring JJ, Fuhler GM, Konstantinov SR, Peppelenbosch MP, Kuipers EJ, de Haar C, van der Woude CJ. Genomic ATG16L1 risk allele-restricted Paneth cell ER stress in quiescent Crohn’s disease. Gut. 2014;63(7):1081–1091. doi:10.1136/gutjnl-2012-303527. 23964099

[8] Raju D, Hussey S, Ang M, Terebiznik MR, Sibony M, Galindo–Mata E, Gupta V, Blanke SR, Delgado A, Romero–Gallo J, et al. Vacuolating cytotoxin and variants in Atg16L1 that disrupt autophagy promote Helicobacter pylori infection in humans. Gastroenterology. 2012;142(5):1160–1171. doi:10.1053/j.gastro.2012.01.043. 22333951 PMC3336037

[9] Wang YH, Gorvel JP, Chu YT, Wu JJ, Lei HY, Ahmed N. Helicobacter pylori impairs murine dendritic cell responses to infection. PLoS One. 2010;5(5):e10844. doi:10.1371/journal.pone.0010844. 20523725 PMC2877707

[10] Wang YH, Wu JJ, Lei HY. The autophagic induction in Helicobacter pylori-infected macrophage. Exp Biol Med (Maywood). 2009;234(2):171–180. doi:10.3181/0808-RM-252. 19064937

[11] Varma M, Kadoki M, Lefkovith A, Conway KL, Gao K, Mohanan V, Tusi BK, Graham DB, Latorre IJ, Tolonen AC, et al. Cell Type- and Stimulation-Dependent Transcriptional Programs Regulated by Atg16L1 and Its Crohn’s Disease Risk Variant T300A. J Immunol. 2020;205(2):414–424. doi:10.4049/jimmunol.1900750. 32522834 PMC7364322

[12] Burada F, Ciurea ME, Nicoli R, Streata I, Vilcea ID, Rogoveanu I, Ioana M. ATG16L1 T300A Polymorphism is Correlated with Gastric Cancer Susceptibility. Pathol Oncol Res. 2016;22(2):317–322. doi:10.1007/s12253-015-0006-9. 26547861

[13] Castano-Rodriguez N, Kaakoush NO, Goh KL, Fock KM, Mitchell HM. Autophagy in Helicobacter pylori Infection and Related Gastric Cancer. Helicobacter. 2015;20(5):353–369. doi:10.1111/hel.12211. 25664588

[14] White E, DiPaola RS. The double-edged sword of autophagy modulation in cancer. Clin Cancer Res. 2009;15(17):5308–5316. doi:10.1158/1078-0432.CCR-07-5023. 19706824 PMC2737083

[15] Oakes SA. Endoplasmic Reticulum Stress Signaling in Cancer Cells. Am J Pathol. 2020;190(5):934–946. doi:10.1016/j.ajpath.2020.01.010. 32112719 PMC7237829

[16] Siwecka N, Rozpedek W, Pytel D, Wawrzynkiewicz A, Dziki A, Dziki Ł, Diehl JA, Majsterek I. Dual role of Endoplasmic Reticulum Stress-Mediated Unfolded Protein Response Signaling Pathway in Carcinogenesis. Int J Mol Sci. 2019;20(18):4354. doi:10.3390/ijms20184354. PMC6770252 31491919

[17] Yadav RK, Chae SW, Kim HR, Chae HJ. Endoplasmic reticulum stress and cancer. J Cancer Prev. 2014;19(2):75–88. doi:10.15430/JCP.2014.19.2.75. 25337575 PMC4204165

[18] Wang Y, Wang JH, Zhang XL, Wang XL, Yang L. Endoplasmic reticulum chaperone glucose-regulated protein 78 in gastric cancer: an emerging biomarker. Oncol Lett. 2018;15(5):6087–6093. doi:10.3892/ol.2018.8114. 29616092 PMC5876464

[19] Ogawa H, Kaira K, Takahashi K, Shimizu A, Altan B, Yoshinari D, Asao T, Oyama T. Prognostic role of BiP/GRP78 expression as ER stress in patients with gastric adenocarcinoma. Cancer Biomark. 2017;20(3):273–281. doi:10.3233/CBM-170062. 28854502

[20] Ikram MA, Brusselle GGO, Murad SD, van Duijn CM, Franco OH, Goedegebure A, Klaver CCW, Nijsten TEC, Peeters RP, Stricker BH, et al. The Rotterdam Study: 2018 update on objectives, design and main results. Eur J Epidemiol. 2017;32(9):807–850. doi:10.1007/s10654-017-0321-4. 29064009 PMC5662692

[21] Buczylko K, Kowalczyk J, Zeman K, Kardas-Sobantka D, Fiszer A. Allergy to food in children with pollinosis. Rocz Akad Med Bialymst. 1995;40:568–572. 8775308

[22] Ding W-X, Ni H-M, Gao W, Hou Y-F, Melan MA, Chen X, Stolz DB, Shao Z-M, Yin X-M. Differential effects of endoplasmic reticulum stress-induced autophagy on cell survival. J Biol Chem. 2007;282(7):4702–4710. doi:10.1074/jbc.M609267200. 17135238

[23] Ohyauchi M, Imatani A, Yonechi M, Asano N, Miura A, Iijima K, Koike T, Sekine H, Ohara S, Shimosegawa T. . The polymorphism interleukin 8 −251 A/T influences the susceptibility of Helicobacter pylori related gastric diseases in the Japanese population. Gut. 2005;54(3):330–335. doi:10.1136/gut.2003.033050. 15710978 PMC1774396

[24] Sorbara MT, Ellison LK, Ramjeet M, Travassos L, Jones N, Girardin S, Philpott D. The protein ATG16L1 suppresses inflammatory cytokines induced by the intracellular sensors Nod1 and Nod2 in an autophagy-independent manner. Immunity. 2013;39(5):858–873. doi:10.1016/j.immuni.2013.10.013. 24238340

[25] Yue C, Li J, Jin H, Hua K, Zhou W, Wang Y, Cheng G, Liu D, Xu L, Chen Y, et al. Autophagy Is a Defense Mechanism Inhibiting Invasion and Inflammation During High-Virulent Haemophilus parasuis Infection in PK-15 Cells. Front Cell Infect Microbiol. 2019;9:93. doi:10.3389/fcimb.2019.00093. 31106159 PMC6499186

[26] Glubb DM, Gearry RB, Barclay ML, Roberts RL, Pearson J, Keenan JI, McKenzie J, Bentley RW . NOD2 and ATG16L1 polymorphisms affect monocyte responses in Crohn’s disease. World J Gastroenterol. 2011;17(23):2829–2837. doi:10.3748/wjg.v17.i23.2829. 21734790 PMC3120942

[27] Balkwill F. Tumour necrosis factor and cancer. Nat Rev Cancer. 2009;9(5):361–371. doi:10.1038/nrc2628. 19343034

[28] Rosentul DC, Plantinga TS, Farcas M, Oosting M, Hamza OJM, Scott WK, Alexander BD, Yang JC, Laird GM, Joosten LAB, et al. Role of autophagy genetic variants for the risk of Candida infections. Med Mycol. 2014;52(4):333–341. doi:10.1093/mmy/myt035. 24713404 PMC4687479

[29] Salem M, Nielsen OH, Nys K, Yazdanyar S, Seidelin JB. Impact of T300A Variant of ATG16L1 on Antibacterial Response, Risk of Culture Positive Infections, and Clinical Course of Crohn’s Disease. Clin Transl Gastroenterol. 2015;6(11):e122. doi:10.1038/ctg.2015.47. 26673830 PMC4816087

[30] Traish AM, Muller RE, Wotiz HH. Interaction of cyproterone acetate with rat prostatic androgen receptors. Steroids. 1985;45(3–4):247–262. doi:10.1016/0039-128X(85)90074-1. 2939604

[31] Ruela-de-sousa RR, Fuhler GM, Blom N, Ferreira CV, Aoyama H, Peppelenbosch MP. Cytotoxicity of apigenin on leukemia cell lines: implications for prevention and therapy. Cell Death Dis. 2010;1(1):e19. doi:10.1038/cddis.2009.18. 21364620 PMC3032507

[32] Yun CW, Lee SH. The Roles of Autophagy in Cancer. Int J Mol Sci. 2018;19(11):3466. doi:10.3390/ijms19113466. PMC6274804 30400561

[33] Mathew R, Karantza-Wadsworth V, White E. Role of autophagy in cancer. Nat Rev Cancer. 2007;7(12):961–967. doi:10.1038/nrc2254. 17972889 PMC2866167

[34] Kang MR, Kim MS, Oh JE, Kim YR, Song SY, Kim SS, Ahn CH, Yoo NJ, Lee SH . Frameshift mutations of autophagy-related genes ATG2B, ATG5, ATG9B and ATG12 in gastric and colorectal cancers with microsatellite instability. J Pathol. 2009;217(5):702–706. 19197948 10.1002/path.2509

[35] Lee JW, Jeong EG, Lee SH, Yoo NJ, Lee SH. Somatic mutations of BECN1, an autophagy-related gene, in human cancers. APMIS. 2007;115:750–756. 17550384 10.1111/j.1600-0463.2007.apm_640.x

[36] Tan J, Fu L, Chen H, Guan J, Chen Y, Fang J. Association study of genetic variation in the autophagy lysosome pathway genes and risk of eight kinds of cancers. Int J Cancer. 2018;143:80–87. 29388190 10.1002/ijc.31288

[37] Mayerle J, Den Hoed CM, Schurmann C, Stolk CL, Homuth G, Peters MJ, Capelle LG, Zimmermann K, Rivadeneira F, Gruska S, et al. Identification of genetic loci associated with Helicobacter pylori serologic status. JAMA. 2013;309(18):1912–1920. 23652523 10.1001/jama.2013.4350

[38] Moazeni-Roodi A, Tabasi F, Ghavami S, Hashemi M. Investigation of ATG16L1 rs2241880 Polymorphism with Cancer Risk: a Meta-Analysis. Medicina (Kaunas). 2019;55(8):425. 10.3390/medicina55080425 PMC6722794 31370304

[39] Diamanti MA, Gupta J, Bennecke M, De Oliveira T, Ramakrishnan M, Braczynski AK, Richter B, Beli P, Hu Y, Saleh M, et al. IKKalpha controls ATG16L1 degradation to prevent ER stress during inflammation. J Exp Med. 2017;214(2):423–437. doi:10.1084/jem.20161867. 28082356 PMC5294863

[40] Fritz T, Niederreiter L, Adolph T, Blumberg RS, Kaser A. Crohn’s disease: NOD2, autophagy and ER stress converge. Gut. 2011;60(11):1580–1588. doi:10.1136/gut.2009.206466. 21252204 PMC3897479

[41] Tschurtschenthaler M, Adolph TE, Ashcroft JW, Niederreiter L, Bharti R, Saveljeva S, Bhattacharyya J, Flak MB, Shih DQ, Fuhler GM, et al. Defective ATG16L1-mediated removal of IRE1alpha drives Crohn’s disease-like ileitis. J Exp Med. 2017;214(2):401–422. doi:10.1084/jem.20160791. 28082357 PMC5294857

[42] Jostins L, Ripke S, Weersma RK, Duerr RH, McGovern DP, Hui KY, Lee JC, Philip Schumm L, Sharma Y, Anderson CA, et al. Host–microbe interactions have shaped the genetic architecture of inflammatory bowel disease. Nature. 2012;491(7422):119–124. doi:10.1038/nature11582. 23128233 PMC3491803

[43] Yahiro K, Satoh M, Nakano M, Hisatsune J, Isomoto H, Sap J, Suzuki H, Nomura F, Noda M, Moss J, et al. Low-density lipoprotein receptor-related protein-1 (LRP1) mediates autophagy and apoptosis caused by Helicobacter pylori VacA. J Biol Chem. 2012;287(37):31104–31115. doi:10.1074/jbc.M112.387498. 22822085 PMC3438942

[44] Bravo J, Diaz P, Corvalan AH, Quest AFG. A Novel Role for Helicobacter pylori Gamma-Glutamyltranspeptidase in Regulating Autophagy and Bacterial Internalization in Human Gastric Cells. Cancers (Basel). 2019;11(6):801. doi:10.3390/cancers11060801. PMC6627848 31185677

[45] Ohata H, Kitauchi S, Yoshimura N, Mugitani K, Iwane M, Nakamura H, Yoshikawa A, Yanaoka K, Arii K, Tamai H, et al. Progression of chronic atrophic gastritis associated with Helicobacter pylori infection increases risk of gastric cancer. Int J Cancer. 2004;109(1):138–143. doi:10.1002/ijc.11680. 14735480

[46] Huang KK, Ramnarayanan K, Zhu F, Srivastava S, Xu C, Tan ALK, Lee M, Tay S, Das K, Xing M, et al. Genomic and Epigenomic Profiling of High-Risk Intestinal Metaplasia Reveals Molecular Determinants of Progression to Gastric Cancer. Cancer Cell. 2018;33(1):137–150 e135. doi:10.1016/j.ccell.2017.11.018. 29290541

[47] Cubillos-Ruiz JR, Bettigole SE, Glimcher LH. Tumorigenic and Immunosuppressive Effects of Endoplasmic Reticulum Stress in Cancer. Cell. 2017;168(4):692–706. doi:10.1016/j.cell.2016.12.004. 28187289 PMC5333759

[48] International Multiple Sclerosis Genetics C. A systems biology approach uncovers cell-specific gene regulatory effects of genetic associations in multiple sclerosis. Nat Commun. 2019;10(1):2236. doi:10.1038/s41467-019-09773-y. 31110181 PMC6527683

[49] Kumar S, Kumari N, Mittal RD, Mohindra S, Ghoshal UC. Association between pro-(IL-8) and anti-inflammatory (IL-10) cytokine variants and their serum levels and H. pylori-related gastric carcinogenesis in northern India. Meta Gene. 2015;6:9–16. doi:10.1016/j.mgene.2015.07.008. 26380815 PMC4556814

[50] Li ZW, Wu Y, Sun Y, Liu LY, Tian MM, Feng GS, You WC, Li JY, et al. Inflammatory cytokine gene polymorphisms increase the risk of atrophic gastritis and intestinal metaplasia. World J Gastroenterol. 2010;16(14):1788–1794. doi:10.3748/wjg.v16.i14.1788. 20380014 PMC2852830

[51] Siregar GA, Halim S, Sitepu VR. Serum TNF-a, IL-8, VEGF levels in Helicobacter pylori infection and their association with degree of gastritis. Acta Med Indones. 2015;47:120–126. 26260554

[52] Fu H, Ma Y, Yang M, Zhang C, Huang H, Xia Y, Lu L, Jin W, Cui D. Persisting and Increasing Neutrophil Infiltration Associates with Gastric Carcinogenesis and E-cadherin Downregulation. Sci Rep. 2016;6(1):29762. doi:10.1038/srep29762. 27412620 PMC4944193

[53] Montfort A, Colacios C, Levade T, Andrieu-Abadie N, Meyer N, Segui B. The TNF Paradox in Cancer Progression and Immunotherapy. Front Immunol. 2019;10:1818. doi:10.3389/fimmu.2019.01818. 31417576 PMC6685295

[54] Crisan TO, Plantinga TS, van de Veerdonk FL, Farcaş MF, Stoffels M, Kullberg BJ, van der Meer JWM, Joosten LAB, Netea MG. et al. Inflammasome-independent modulation of cytokine response by autophagy in human cells. PLoS One. 2011;6(4):e18666. doi:10.1371/journal.pone.0018666. 21490934 PMC3072416

[55] Jiang GM, Tan Y, Wang H, Peng L, Chen H-T, Meng X-J, Li -L-L, Liu Y, Li W-F, Shan H, et al. The relationship between autophagy and the immune system and its applications for tumor immunotherapy. Mol Cancer. 2019;18(1):17. doi:10.1186/s12943-019-0944-z. 30678689 PMC6345046

[56] Ghayur T, Banerjee S, Hugunin M, Butler D, Herzog L, Carter A, Quintal L, Sekut L, Talanian R, Paskind M, et al. Caspase-1 processes IFN-gamma-inducing factor and regulates LPS-induced IFN-gamma production. Nature. 1997;386(6625):619–623. doi:10.1038/386619a0. 9121587

[57] Yamauchi K, Choi IJ, Lu H, Ogiwara H, Graham DY, Yamaoka Y. Regulation of IL-18 in Helicobacter pylori infection. J Immunol. 2008;180(2):1207–1216. doi:10.4049/jimmunol.180.2.1207. 18178861 PMC2827480

[58] Majima T, Ichikura T, Chochi K, Kawabata T, Tsujimoto H, Sugasawa H, Kuranaga N, Takayama E, Kinoshita M, Hiraide H, et al. Exploitation of interleukin-18 by gastric cancers for their growth and evasion of host immunity. Int J Cancer. 2006;118(2):388–395. doi:10.1002/ijc.21334. 16049975

[59] Den Hoed CM, Holster IL, Capelle LG, de Vries AC, den Hartog B, Ter Borg F, Biermann K, Kuipers EJ, et al. Follow-up of premalignant lesions in patients at risk for progression to gastric cancer. Endoscopy. 2013;45(4):249–256. 23533073 10.1055/s-0032-1326379

[60] Den Hollander WJ, Holster IL, Den Hoed CM, Capelle LG, Tang TJ, Anten MP, Prytz-Berset I, Witteman EM, Ter Borg F, den Hartog G, et al. Surveillance of premalignant gastric lesions: a multicentre prospective cohort study from low incidence regions. Gut. 2019;68(4):585–593. doi:10.1136/gutjnl-2017-314498. 29875257

[61] Bartfeld S, Bayram T, van de Wetering M, Huch M, Begthel H, Kujala P, Vries R, Peters PJ, Clevers H. In vitro expansion of human gastric epithelial stem cells and their responses to bacterial infection. Gastroenterology. 2015;148(1):126–136 e126. doi:10.1053/j.gastro.2014.09.042. 25307862 PMC4274199

[62] Phillips T, Murray G, Wakamiya K, Askaa J, Huang D, Welcher R, Pii K, Allred DC. Development of standard estrogen and progesterone receptor immunohistochemical assays for selection of patients for antihormonal therapy. Appl Immunohistochem Mol Morphol. 2007;15(3):325–331. doi:10.1097/01.pai.0000213135.16783.bc. 17721279

[63] Gunaletchumy SP, Teh X, Khosravi Y, Ramli NSK, Chua EG, Kavitha T, Mason JN, Lee HT, Alias H, Zaidan NZ, et al. Draft genome sequences of Helicobacter pylori isolates from Malaysia, cultured from patients with functional dyspepsia and gastric cancer. J Bacteriol. 2012;194(20):5695–5696. doi:10.1128/JB.01278-12. 23012278 PMC3458671

[64] Hoekstra E, Das AM, Willemsen M, Swets M, Kuppen PJK, van der Woude CJ, Bruno MJ, Shah JP, Hagen TLMT, Chisholm JD, et al. Lipid phosphatase SHIP2 functions as oncogene in colorectal cancer by regulating PKB activation. Oncotarget. 2016;7(45):73525–73540. doi:10.18632/oncotarget.12321. 27716613 PMC5341996

