# Supplementary material for: Autophagy mediates ER stress and inflammation in Helicobacter pylori-related gastric cancer
Source: Gut Microbes. 2021 Dec 29;14(1):2015238. doi: 10.1080/19490976.2021.2015238 (PMC8726742; doi:10.1080/19490976.2021.2015238)
Supplement: Supplemental Material [file KGMI_A_2015238_SM2233.zip › supplementary/Supplementary Material_restructured_Final revision_clean.docx]

Supplementary material belonging to

**Autophagy mediates ER stress and inflammation in *Helicobacter pylori*-related gastric cancer**

**M.C. Mommersteeg^1^*, I. Simovic^2*^,** B. Yu^1^, S.A.V. van Nieuwenburg^1^, I, M.J. Bruno^1^, G.L.Porras-Hurtado, H.A. Salazar-Carmona, A.R. Cobo-Alvarado, J. L. Cardona-Deazza, M. Doukas^3^, E.J. Kuipers^1^, M.C.W. Spaander^1^**,** M.P. Peppelenbosch^1^, **N. Castaño Rodriguez^2#^, G.M. Fuhler^1#^**

**Supplementary Materials and methods**

**Patient selection**

Dutch patients presenting with gastric premalignant lesions were included from the ongoing prospective Progression or regression of gastric premalignant lesions of the stomach (Proregal) cohort study^1, 2^ (Table S1). In this cohort, patients who have been diagnosed with any gastric premalignant lesions are selected and followed. Patients are excluded if they are under the age of 18, above the age of 75, have previously undergone surgery of the upper gastrointestinal tract, have a diagnosis of any malignancy not considered in remission or have a proven *CDH1* mutation. Patients undergo surveillance at 1 and 3 years after the baseline endoscopy. Following the second endoscopy, surveillance is performed according to current clinical guidelines^3^. At each endoscopy, 12 biopsies are taken (4 antrum, 2 angulus, 2 greater curvature, 2 lesser curvature and 2 cardia). Biopsies are histopathologically examined by an expert pathologist and graded according to the operative link on gastric intestinal metaplasia assessment (OLGIM) staging system^4^.

*H. pylori* infection status in all subjects within the Australian population was determined serologically using the Helico Blot 2.1 Kit (MP Biomedicals, Sydney, Australia). Disease was confirmed via histopathological examination by expert pathologists. Exclusion criteria in this population included known infection with the human immunodeficiency virus (HIV), any comorbidity associated with immunosuppression, a recent history (< 3 months) of prescription of antimicrobial agents, nonsteroidal anti- inflammatory drugs (NSAIDs), or acid suppressants. All subjects provided written informed consent to participate in the study.

### Human genomic DNA isolation and genotyping

For the Dutch IM population, DNA was isolated from whole blood using the Kleargene XL blood DNA extraction kit (LGC limited, Teddington, UK). Quantity and quality of isolated DNA was measured by spectophotometry (Nanodrop 2000, Thermofischer scientific, Waltham, United states) and DNA samples were normalized to 10ng/ul. *ATG16L1* rs2241880 was determined using Polymerase Chain Reaction-Restriction Fragment Length Polymorphism (PCR-RFLP). In short, DNA fragments were amplified using a regular PCR (primer sequences and PCR protocols can be found in the supplements). Amplicons that provided a well-defined band were further digested overnight using the restriction enzyme BufI (Thermofischer scientific) that specifically digests the A allele. The digested samples were then visualised by TBE gel electrophoresis and genotypes were determined on basis of the fragment lengths (see **Figure S3** for examples).

For the Australian population, genomic DNA was extracted from whole peripheral blood samples using the QIAamp Blood Mini Kit (Qiagen, Melbourne, Australia) according to the manufacturer’s instructions. Samples were then standardised to 10 ng/ul for customised genotyping using matrix-assisted laser-desorption ionisation time-of-flight (MALDI-TOF) mass spectrometry (MS), and the Agena Bioscience MassARRAY iPLEX assay (San Diego, USA), at the Australian Genome Research Facility Ltd, St Lucia, University of Queensland, Australia. As a method of quality control and to validate the MALDI-TOF genotyping results, 10% of samples from this population were randomly selected to be genotyped via Sanger sequencing and fragment analysis.

### Cell lines

The GC epithelial cell line SK-GT-2 was cultured in RPMI 1640 medium (Thermofischer) supplemented with 5% foetal calf serum (FCS) and 1% penicillin/streptomycin (P/S). The immortalized gastric epithelial cell line GES-1 was cultured in DMEM (Thermofischer) supplemented with 10% FCS and 1% P/S. The human GC cell line AGS (American Type Culture Collection (ATCC), Manassas, USA; code: ATCC CRL-1739), was cultured in F-12 K media (Gibco, Sydney, Australia), supplemented with 10% FCS (Bovogen, Victoria, Australia) and 100 ug/mL P/S (Gibco, Sydney, Australia).

### CRISPR/Cas9 Genome Editing

CRISPR/Cas9 genome editing technology was implemented to produce *ATG16L1* rs2241880 knock-in AGS cell lines representing all three genotypes of the polymorphism (AA, AG, GG). Design and selection of the appropriate target site and synthetic short guide RNA (sgRNA) for homology-directed repair (HDR) was carried out using available online tools including CHOPCHOP version 2.0^5^ and E-CRISP version 5.3^6^. The synthetic sgRNA (5’ -CTCACTTCTTTACCAGAACC – ‘3) was supplied by Synthego (Menlo Park, USA), while the polymorphism-containing single stranded oligodeoxynucleotide (ssODN) donor template (5’-CTGAAGCATACTTACGAAGACACACAAGGCAGTAGCTGGTACCCTCACTTCTTTACCAGAACCAGTATGAGCATCCACATTGTCCTGGGGGACTGGGAAGGAAGAGACAGAGCGTCTCCT – ‘3) was supplied by Integrated DNA technologies (Coralville, USA). AGS cells were seeded in 24-well plates at a concentration of 8 x 10^4^ cells/ml with antibiotic-free cell culture media until a confluency of 70 % was achieved. Transfection of the CRISPR machinery and the GeneArt Platinum Cas9 Nuclease (Thermo Fisher Scientific, Sydney, Australia) was performed using the Lipofectamine CRISPRMAX kit (Thermo Fisher, Sydney, Australia) according to the manufacturer’s instructions. Following transfection, cells were sorted for clonal selection and expansion using the BD FACS Aria II cell sorter.

**Organoids**

Stomach organoids were cultured as previously published^7^. In brief, biopsies from both antrum and corpus were collected from patients included in the previously described Proregal cohort. These patients were aged between 41 and 78, none were *H. Pylori* positive at the time of biopsy, all had intestinal metaplasia in at least one compartment of the stomach. These biopsies were washed, minced and collagenized using collagenase type A (Sigma-Aldrich). Cells were subsequently seeded in matrigel (Corning, New York, United States). Cells were maintained in either expansion or differentiation medium containing: Wnt3A conditioned medium (expansion medium only), Noggin conditioned medium, R-Spondin conditioned medium, FGF10 (Peprotech, Londen, United Kingdom), EGF (Peprotech), B27 (Invitrogen), Gastrin (Sigma-Aldrich), TGF-βi (A-83-01, Bristol, United Kingdom), Nicotinamide (Sigma-Aldrich, only expansion medium) and RHOKi (Y-27632, Sigma-Aldrich, only during initiation). Organoid medium was replaced three times a week and cultures were split approximately every two weeks (**Figure S4A, C and E**).

Organoids were investigated using basic immunohistochemistry like hemotoxilin eosin stains as well as Alcian Blue PAS stains to evaluate the mucin profile (**Figure S4B and D)**. For this purpose, formalin fixed organoids were embedded in paraffin and sections were deparafinized in sequence of ethanols and rehydrated in demineralized water (dH2O). Slides were incubated for 30 minutes in alcian blue solution and rinsed in dH2O, alcian blue stained slides were oxidized in 0.5% periodic acid and washed in dH2O after which they were incubated in Schiff’s reagent for 20 minutes, dehydrated and pertex mounted. Immunohistochemistry to show G-cells was performed as it has been described previously in this paper, using a gastrin primary antibody (DAKO # GA519). A small number of cells in the organoids are positive for gastrin as shown in **Figure S4F**. RT-PCR was performed for several gastric markers including gastric (Muc5ac and Muc6) and intestinal (Muc2) mucins, showing all organoids being positive for both gastric mucins (**Figure S4G**). The primers and protocols used are outlined in **Tables S3** and **S4**.

### Immunohistochemistry

Immunohistochemistry was performed as described previously^7^ on 5µm slices of formalin fixed paraffin embedded antral biopsies. After deparafinization and antigen retrieval using heated citric acid, slides were blocked using normal goat serum (Vector laboratories, Peterborough, United Kingdom). The GRP78 rabbit monoclonal antibody (#3177S, Cell signalling technology, Danvers, United States) was incubated overnight at 4°C. EnVision anti-rabbit-HRP (Agilent, Santa Clara, United States) was used as secondary antibody and antibody binding was visualized by di-amino-benzidine tetrahydrochloride hydrate (Sigma-Aldrich) and haematoxylin (O-Kindler, Freiburg, Germany). Positivity for GRP78 was quantified using the Allredscore.

***Helicobacter pylori* culture and infection assays**

*H. pylori* strain 43504 (*cagA^+^*, *vacA^+(S1M1)^*) was acquired from ATCC (Wesel, Germany). The *H. pylori* strain GC026 (*cag*A*^+^. cag*E*^+^, cag*L*^+^, cag*T*^+^, vacA^+^* ^s1m1^, *bab*A^+^, *oip*A^+^, *dupA*^+^, and *sabA*^+^) was previously isolated from a Malaysian GC patient^28^. The presence of HP-16S, *UreA*, *VacA* and *CagA* was used to assess culture conditions and bacterial viability (**Table S2**). For assessment of ER stress and autophagy markers in GES-1 and SK-GT-2 using western blot analysis, and for IL-8 production of peripheral blood mononuclear cells (PBMCs), *H. pylori* strain 43504 was harvested, heat killed for 10 minutes at 70°C + 5 minutes at 95°C and used at a concentration of 1e7 CFU/ml in stimulation experiments. As control, *E. coli* (strain DH5α) was used. For infection of *ATG16L1* rs2241880 knock-in AGS cell lines, cells were seeded at a concentration of 5 x 10^5^ cells/ml in 24-well plates and incubated overnight to develop a monolayer. Live *H. pylori* strain GC026 was added to achieve a multiplicity of infection (MOI) of 100. At 24 hours, RNA extraction was conducted using the Isolate II RNA Extraction Kit (Bioline, Sydney, Australia) and cDNA was synthesised using the SensiFast cDNA synthesis kit (Bioline, Sydney, Australia), following the manufacturers’ protocols.

### Western blot analysis

Western blot was performed as described previously^8^. Cells were treated as described in the main text. Tunicamycin was obtained from Sigma-Aldrich (Saint Louis, United States). Protein lysates from gastric cells were prepared by lysing phosphate buffered saline (PBS, Gibco)-washed cells in Laemmli buffer 2x (Tris-HCl (pH 6.8) 100 mM, dithiothreitol 200 mM, 4% SDS, 20% glycerol and 0,1% bromophenol blue) and were further heated for 10 min at 95⁰C. Alternatively, an *in-house* prepared RIPA buffer (50 mM Tris-base, 150 mM NaCl, 1mM EDTA, 0.1% SDS, 0.5% sodium deoxycholate, 1% Triton x-100/NP-40, adjusted to pH 7.4) supplemented with cOmplete^TM^ Protease Inhibitor Cocktail (Roche) was used for protein lysate extraction. In this case, after lysis, samples were further prepared by adding 20 ug of protein lysate to Laemmli 2x Concentrate sample buffer (Sigma) in a 1:1 volume ratio and boiled for 5 minutes at 95⁰C. Cell lysates were loaded on acrylamide/bis-acrylamide based gel or 10% Mini-PROTEAN TGX^TM^ Precast Protein Gel (BioRad). Proteins were separated by SDS-PAGE and subsequently transferred to Immobilon-P (Sigma-Aldrich) or Immuno-Blot^TM^ PVDF (0.2 um, BioRad) membranes. Blocking was performed in 1:1 diluted odyssey blocking buffer (LI-COR Biosciences, Lincoln, NE, USA) with PBS or 5% Skim milk powder in PBS-Tween. Incubation with the primary antibodies (GRP78 #3177S, LC3B-I/II #2775S, p62 #5114S; Cell Signalling Technology) was performed overnight at 4°C. After washing in PBS-Tween (PBS with 0.5% Tween 20), membranes were incubated with the secondary IRDye antibodies (LI-COR Biosciences, Lincoln, NE, USA) or Goat Anti-Rabbit IgG (H+L)-HRP Conjugate (BioRad), for 1h. Detection was performed using Odyssey reader and analyzed using the manufacturer’s software or the ImageQuant LAS 500 (GE Life Sciences, Uppsala, Sweden) and analyzed using ImageJ (v1.52a) (National Institutes of Health, USA). All target proteins were normalized to B-Actin (C4) (Santa Cruz Biotechnology).

**Supplementary Figures**


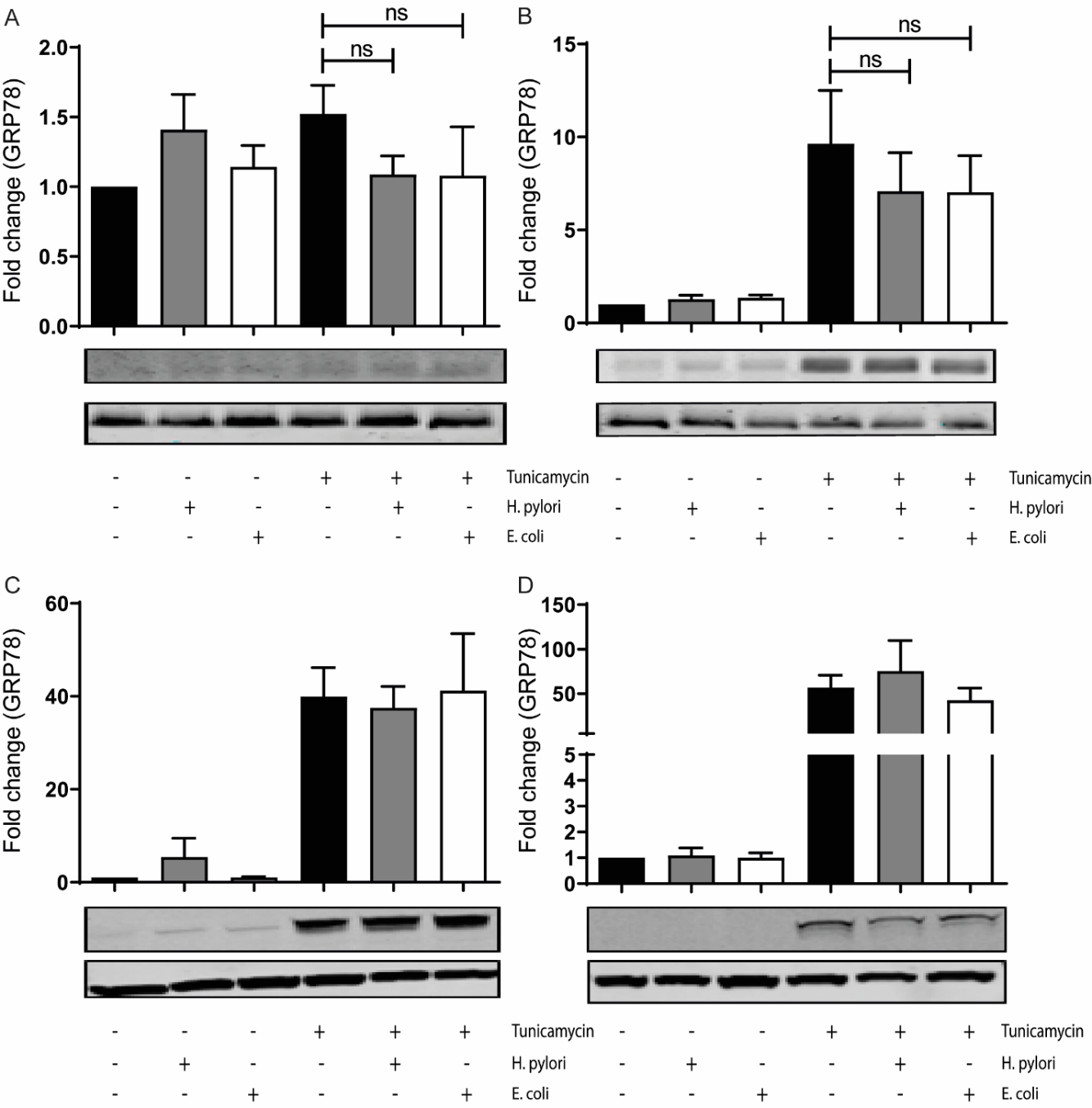


**Figure S1. *H. pylori* does not reduce ER stress levels in esophageal or colon epithelial cells** We investigated if the reduction of ER stress levels upon stimulation with *H. pylori* was specific for the gastric epithelium. We therefore subjected colorectal cancer cell lines (Caco2 and HCT116) and esophageal cancer cell lines (TE1 and TE11) to *H. pylori* or *E. coli* infection in the presence or absence of tunicamycin. Protein expression of GRP78 was measured as an ER stress cell marker**.** GRP78 expression in Caco-2 colorectal cancer cells **(A)**, HCT116 colorectal cancer cells **(B)**, TE1 esophageal cancer cells **(C)** and TE11 esophageal cancer cells **(D)** determined by western blot analysis. Representative example as well as quantification of 4 individual experiments is shown. GRP78 expression relative to B-actin and normalized against unstimulated sample are shown. In these cell types, no significant reduction of GRP78 was observed upon *H. pylori* stimulation.


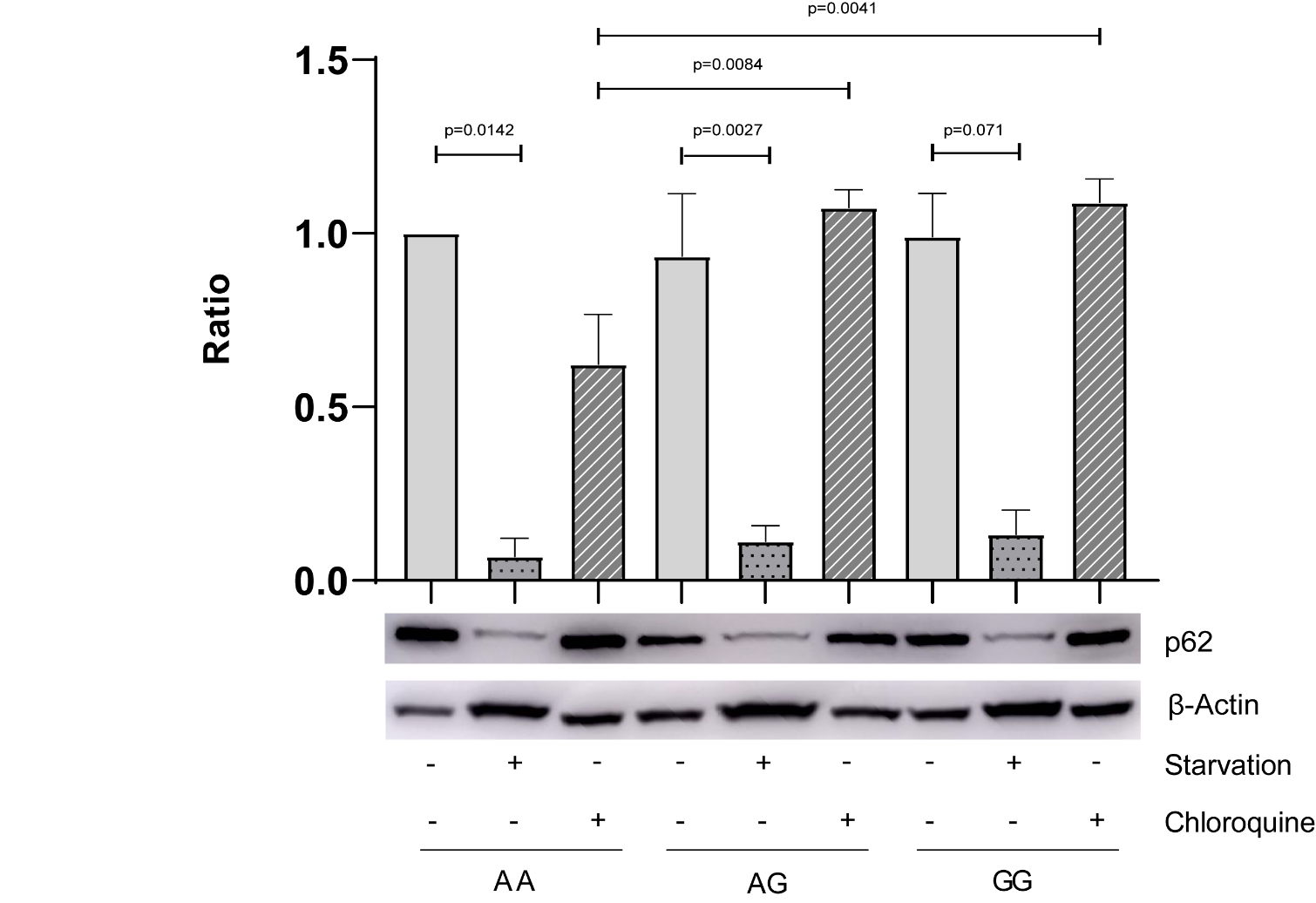


**Figure S2. p62 turnover assays in genetically modified gastric epithelial cells.** Cells were either untreated or treated with starvation or chloroquine for 6 hrs. p62 appears to accumulate in AGS cells treated with chloroquine, indicating autophagy inhibition. Accumulation of p62 is significantly higher in G-carrying cells (AG and GG) compared to AA-carrying cells. On the other hand, starvation leads to a significant decrease of p62 levels in all starved AGS cells, indicating induction of autophagy. However, this decrease appears to be less pronounced in GG-carrying cells when compared to A-carrying cells (AG and AA). Ratios were normalized to the control (i.e. AA-carrying cells, no stimuli). Blots shown are a representative of three independent experiments. AA; wild-type homozygote, AG; heterozygote, GG; mutant homozygote.


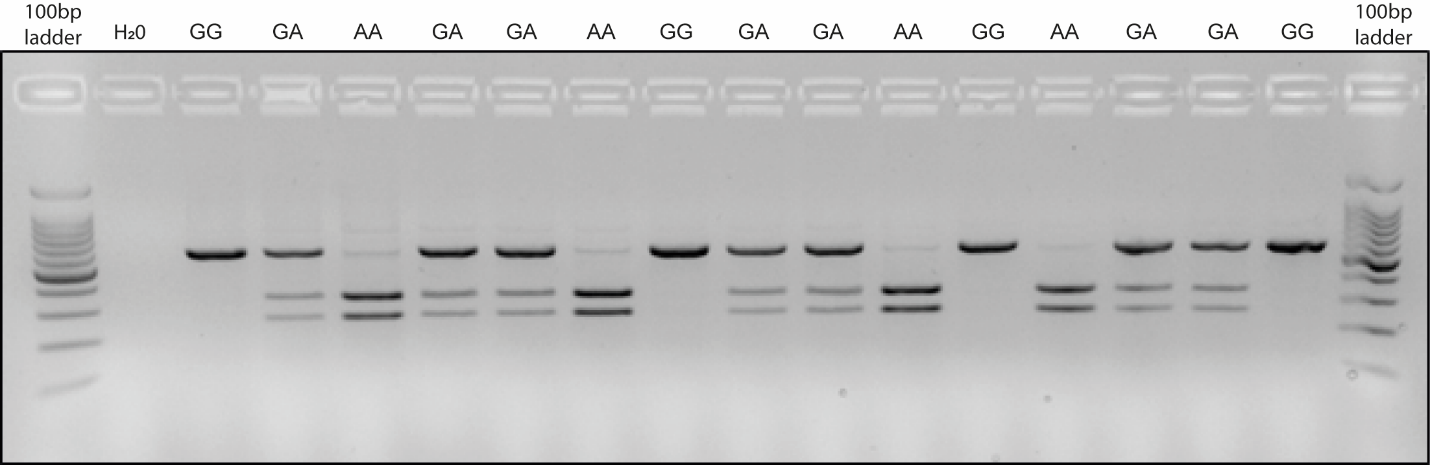


### Figure S3. Examples of PCR-RFLP genotyping for *ATG16L1* rs2241880. PCR amplicons are subjected to restriction enzyme BufI. The A allele is digested by this enzyme, resulting in two shorter fragments, while the G-allele is not. Thus, one ~600 bp band designates the presence of two G alleles, two smaller bands denominates two A alleles, and the presence of three bands indicates heterozygotes. The assay was validated using previously genotyped DNA^9^.


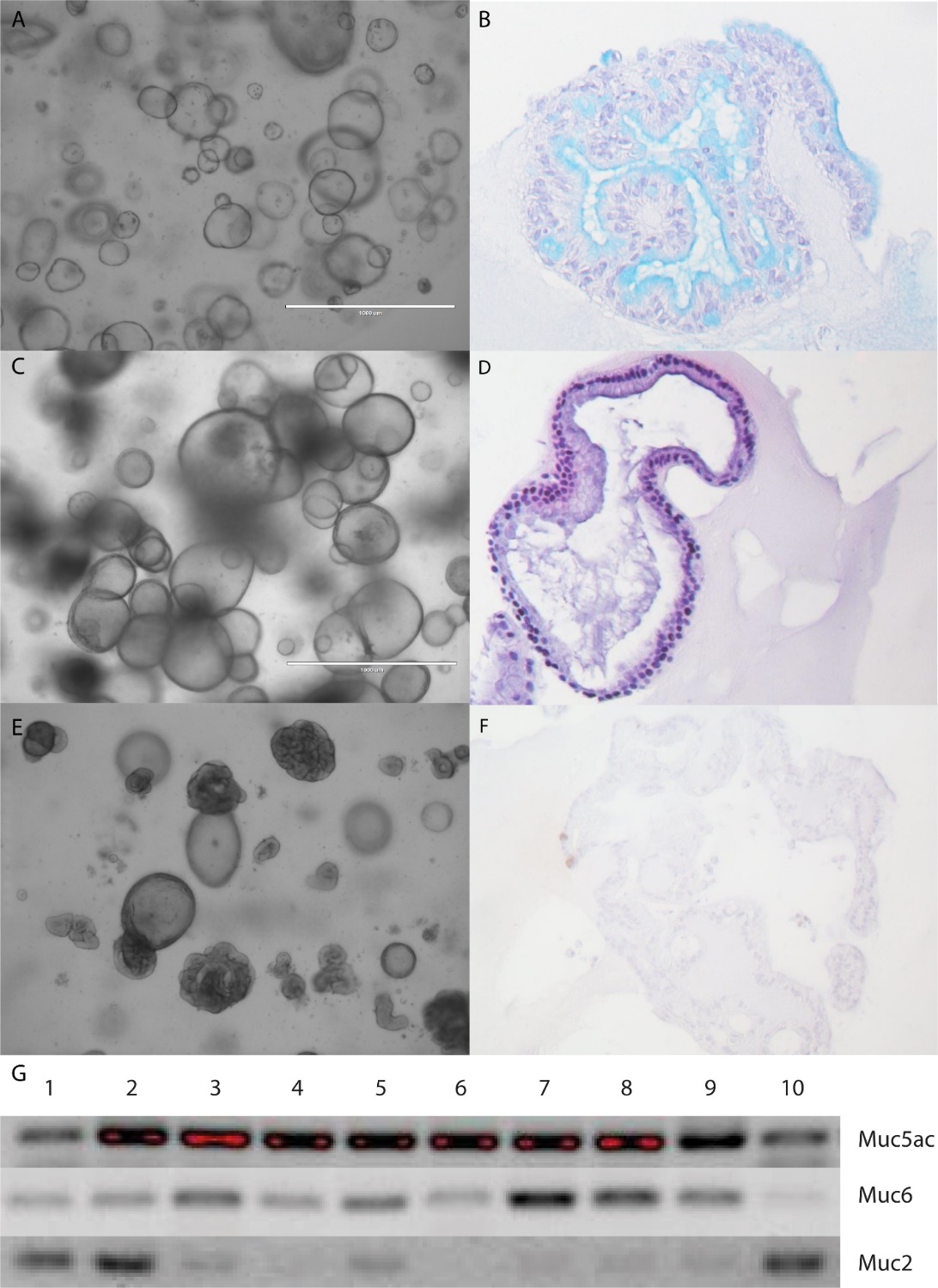


**Figure S4.** **A, C and E:** white light pictures of gastric organoids when confluent, E further enlarged to show morphology. **B:** Alcian blue-PAS stain for mucin evaluation of a gastric organoid. **D:** Hematoxilin/eosin (H&E) stain of a gastric organoid. **F**: immunohistochemistry targeting gastrin to show G-cells in gastric organoids (DAB). **G:** PCR results of several established gastric and intestinal mucins expressed in 10 different gastric organoids.


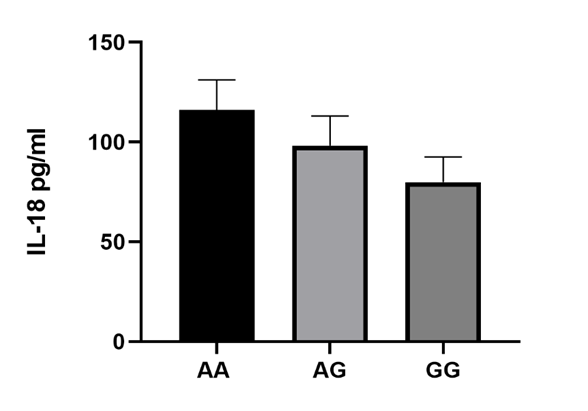


**Figure S5. Concentration of IL-18 in *H. pylori* strain GC0260-challenged edited and non-edited AGS cells.** Experiments were conducted in triplicates. Statistical analysis was carried out using parametric t-test (two-tailed) with error bars representing SEM. AA; wild-type homozygotes, AG; heterozygote, GG; mutant homozygote.

**Supplementary Tables**

**Table S1. Clinical characteristics of the study samples at baseline.**

| **Characteristic** | **Australian**  N = 349 | | **Proregal cohort**  N=308 |
| --- | --- | --- | --- |
|  | Controls*  (N = 232) | GC  (N = 117) | IM |
| **Sex (N)**  **Male/Female** | 154/78 | 77/40 | 148/160 |
| **Age**  **Median ± SD** | 61.4 ± 6.8 | 60.8 ± 6.7 | 61.0 ± 6.3 |
| ***Helicobacter pylori* status (%)**  **Ever Positive**  **Positive**  **Negative**  **Unknown^#^** | -  65.0  33.2  1.8 | -  82.9  15.4  1.7 | 48.0  31.1  52.0  - |

N, number; SD, standard deviation; Atrophy, atrophic gastritis; IM, intestinal metaplasia; GC, gastric cancer. * Controls were healthy individuals. ^#^ Serum to determine *Helicobacter pylori* infection was not available for these individuals.

**Table S2. Characteristics of the primers used for bacterial targets.**

| **Gene** | **Sequence (5’ – 3’)** | **Tm^o^** | **Product Size (bp)** |
| --- | --- | --- | --- |
| *VacA S1/S2* F  *VacA S1/S2* R | ATGGAAATACAACAAACACAC CTGCTTGAATGCGCCAAAC | 57.9  58.6 | 259/286 |
| *UreA* F  *UreA* R | ATG AAA CTC ACC CCA AAA GA TTC ACT TCA AAG AAA TGG AAG TGT GA | 61.2  52.6 | 452 |
| *16S HP* F  *16S HP* R | TTTGTTAGAGAAGATAATGACGGTATCTAAC CATAGGATTTCACACCTGACTGACTAT C | 59.7  61.2 | 154 |
| *CagA* F  *CagA* R | GATAACAGGCAAGCTTTTGAGA  CTGCAAAAGATTGTTTGGCA | 56.9  54.7 | 349 |

Tm, melt temperature; bp, base pairs; F, forward primer; R, reverse primer; *VacA*, Vacuolating cytotoxin; *UreA*, Urease; *16s HP*, 16s ribosomal RNA H. pylori; *CagA*, cytotoxin associated gene A

**Table S3. Characteristics of the primers used for human targets.**

| **Gene** | **Sequence (5’ – 3’)** | **Tm^o^** | **Product Size (bp)** |
| --- | --- | --- | --- |
| *GAPDH* F  *GAPDH* R | ACAGTTGCCATGTAGACC  TTGAGCACAGGGTACTTTA | 55.7  55.8 | 88 |
| *RPLP0* F  *RPLP0* R | GGCGACCTGGAAGTCCAACT  CCATCAGCACCACAGCCTTCC | 67.7  68.2 | 149 |
| *IL8* F  *IL8* R | GAATGGGTTTGCTAGAATGTGATA  CAGACTAGGGTTGCCAGATTTAAC | 62.8  63.7 | 129 |
| *TNFA* F | GAGGCCAAGCCCTGGTATG | 66.8 | 91 |
| *TNFA* R | CGGGCCGATTGATCTCAGC | 70.3 |  |
| *IL18* F | GCTTGAATCTAAATTATCAGTC | 53.9 | 342 |
| *IL18* R | GAAGATTCAAATTGCCATCTTAT | 59.9 |  |
| *IL1B* F | TTCGAGACATGGGATAACGAG | 67.9 | 794 |
| *IL1B* R | TTAGAACCAAATGTGGCCGTG | 68.4 |  |
| *IL10* F | GACTTTAAGGGTTACCTGGGTTG | 63.4 | 112 |
| *IL10* R | TCACATGCGCCTTGATGTCTG | 69.7 |  |
| *IL17A* F | CTCATTGGTGTCACTGCTACTG | 62.4 | 78 |
| *IL17A* R | CCTGGATTTCGTGGGATTGTG | 68.5 |  |
| *G ATG16L1* F  *G ATG16L1* R | TTTCCTTTGCCCCATCCCTC  TAAGGCATGTGCTGGCTCTC | 60.0  60.1 | 571 |
| *Muc2* F  *Muc2* R | AGGATGACACCATCTACCTCAC  CATCGCTCTTCTCAATGAGCA | 58.9  58.5 | 102 |
| *Muc5ac* F  *Muc5ac* R | GGAGGTGCCCACTTCTCAAC  CTTCAGGCAGGTCTCGCTG | 60.6  60.4 | 153 |
| *Muc6* F  *Muc6* R | CAGCTCAACAAGGTGTGTGC  TGGGGAAAGGTCTCCTCGTA | 60.0  59.6 | 149 |

Tm, melt temperature; bp, base pairs; G, genomic primer; F, forward primer; R, reverse primer; *GAPDH*, glyceraldehyde 3-phosphate dehydrogenase; *RPLP0,* ribosomal protein lateral stalk subunit P0*; IL*, interleukin; TNF-α, tumor necrosis factor alpha; *ATG16L1,* autophagy related 16 like 1; Muc2, mucin 2; Muc5ac, mucin 5ac; Muc6, mucin 6

**Table S4. Details for qRT-PCR (protocol 1 - 5), PCR-RFLP (protocol 6) and RT-PCR (protocol 7).**

|  | **Protocol 1** | **Protocol 2** | | **Protocol 3** | | **Protocol 4** | **Protocol 5** | **Protocol 6** | **Protocol 7** |
| --- | --- | --- | --- | --- | --- | --- | --- | --- | --- |
| **Gene** | *RPLP0 / IL10* | | *GAPDH / IL18* | | *IL8* | *TNFA / IL1B* | *IL17A* | *ATG16L1* | *Muc2/Muc5ac/Muc6* |
| **Steps** |  | |  | |  |  |  |  |  |
| Hold  Hold 1  Hold 2  Cycling  Denaturing  Annealing  Extension  Final extension  Melt  Final Hold | 50 ^o^C x 2 mins  95 ^o^C x 10 mins  (40 cycles)  95 ^o^C x 15 secs  60 ^o^C x 30 secs  72 ^o^C x 30 secs  72 ^o^C x 10 mins  50 – 99 ^o^ C  - | | 50 ^o^C x 2 mins  95 ^o^C x 10 mins  (40 cycles)  95 ^o^C x 15 secs  55 ^o^C x 30 secs  72 ^o^C x 30 secs  72 ^o^C x 10 mins  50 – 99 ^o^ C  - | | 95 ^o^C x 10 mins  -  (45 cycles)  95 ^o^C x 10 secs  60 ^o^C x 30 secs  -  -  60 – 95 ^o^C  - | 50 ^o^C x 2 mins  95 ^o^C x 10 mins  (40 cycles)  95 ^o^C x 15 secs  60 ^o^C x 60 secs  -  -  50 – 99 ^o^ C  - | 50 ^o^C x 2 mins  95 ^o^C x 10 mins  (40 cycles)  95 ^o^C x 15 secs  63 ^o^C x 30 secs  72 ^o^C x 30 secs  72 ^o^C x 10 mins  50 – 99 ^o^ C  - | 95 ^o^C x 7 mins  -  (35 cycles)  95 ^o^C x 30 secs  65 ^o^C x 30 secs  72 ^o^C x 60 secs  72 ^o^C x 10 mins  -  4 ^o^C x ∞ mins | 95 ^o^C x 10 mins  -  (35 cycles)  95 ^o^C x 35 secs  55-60 ^o^C x 30 secs  72 ^o^C x 30 secs  72 ^o^C x 10 mins  -  4 ^o^C x ∞ mins |
| Incubation of restriction enzyme (BfuI)  Heat inactivation of restriction enzyme (BfuI)  Final Hold | -  -  - | | -  -  - | | -  -  - | -  -  - | -  -  - | 37 ^o^ C x 360 mins  80 ^o^ C x 10 mins  4 ^o^C x ∞ mins | -  -  - |

*GAPDH*, glyceraldehyde 3-phosphate dehydrogenase; *RPLP0,* ribosomal protein lateral stalk subunit P0; IL, interleukin; TNF-α, tumor necrosis factor alpha; ATG16L1, autophagy related 16 like 1; Muc2, mucin 2; Muc5ac, mucin 5ac; Muc6, mucin 6; mins, minutes; secs, seconds.

**References**

1. den Hollander WJ, Holster IL, den Hoed CM, et al. Surveillance of premalignant gastric lesions: a multicentre prospective cohort study from low incidence regions. Gut 2019;68:585-593.

2. den Hoed CM, Holster IL, Capelle LG, et al. Follow-up of premalignant lesions in patients at risk for progression to gastric cancer. Endoscopy 2013;45:249-56.

3. Pimentel-Nunes P, Libanio D, Marcos-Pinto R, et al. Management of epithelial precancerous conditions and lesions in the stomach (MAPS II): European Society of Gastrointestinal Endoscopy (ESGE), European Helicobacter and Microbiota Study Group (EHMSG), European Society of Pathology (ESP), and Sociedade Portuguesa de Endoscopia Digestiva (SPED) guideline update 2019. Endoscopy 2019;51:365-388.

4. Capelle LG, de Vries AC, Haringsma J, et al. The staging of gastritis with the OLGA system by using intestinal metaplasia as an accurate alternative for atrophic gastritis. Gastrointest Endosc 2010;71:1150-8.

5. Labun K, Montague TG, Gagnon JA, et al. CHOPCHOP v2: a web tool for the next generation of CRISPR genome engineering. Nucleic Acids Res 2016;44:W272-6.

6. Heigwer F, Kerr G, Boutros M. E-CRISP: fast CRISPR target site identification. Nat Methods 2014;11:122-3.

7. Bartfeld S, Bayram T, van de Wetering M, et al. In vitro expansion of human gastric epithelial stem cells and their responses to bacterial infection. Gastroenterology 2015;148:126-136 e6.

8. Hoekstra E, Das AM, Willemsen M, et al. Lipid phosphatase SHIP2 functions as oncogene in colorectal cancer by regulating PKB activation. Oncotarget 2016;7:73525-73540.

9. Deuring JJ, Fuhler GM, Konstantinov SR, et al. Genomic ATG16L1 risk allele-restricted Paneth cell ER stress in quiescent Crohn's disease. Gut 2014;63:1081-91.
